# Light emission enhancement by supramolecular complexation of chemiluminescence probes designed for bioimaging†
†Electronic supplementary information (ESI) available. See DOI: 10.1039/c8sc05174g


**DOI:** 10.1039/c8sc05174g

**Published:** 2019-01-16

**Authors:** Samer Gnaim, Anna Scomparin, Anat Eldar-Boock, Christoph R. Bauer, Ronit Satchi-Fainaro, Doron Shabat

**Affiliations:** a School of Chemistry , Raymond and Beverly Sackler Faculty of Exact Sciences , Israel . Email: chdoron@tauex.tau.ac.il; b Department of Physiology and Pharmacology , Sackler Faculty of Medicine , Tel Aviv University , Tel Aviv 69978 , Israel; c Department of Drug Science and Technology , University of Turin , Via P. Giuria 9 , 10125 Turin , Italy; d Bioimaging Center , University of Geneva , Geneva , Switzerland

## Abstract

Chemiluminescence offers advantages over fluorescence for bioimaging, since an external light source is unnecessary with chemiluminescent agents. This report demonstrates the first encapsulation of chemiluminescence phenoxy-adamantyl-1,2-dioxetane probes with trimethyl β-cyclodextrin. Clear proof for the formation of a 1 : 1 host–guest complex between the adamantyl-1,2-dioxetane probe and trimethyl β-cyclodextrin was provided by mass spectroscopy and NMR experiments. The calculated association constant of this host–guest system, 253 M^–1^, indicates the formation of a stable inclusion complex. The inclusion complex significantly amplified the light emission intensity relative to the noncomplexed probe under physiological conditions. Complexation of adamantyl-dioxetane with fluorogenic dye-tethered cyclodextrin resulted in light emission through energy transfer to a wavelength that corresponds to the fluorescent emission of the conjugated dye. Remarkably, the light emission intensity of this inclusion complex was approximately 1500-fold higher than that of the non-complexed adamantyl-dioxetane guest. We present the first demonstration of microscopic cell images obtained using a chemiluminescence supramolecular dioxetane probe and demonstrate the utility of these supramolecular complexes by imaging of enzymatic activity and bio-analytes *in vitro* and *in vivo*. We anticipate that the described chemiluminescence supramolecular dioxetane probes will find use in various biological applications.

## Abstract 2

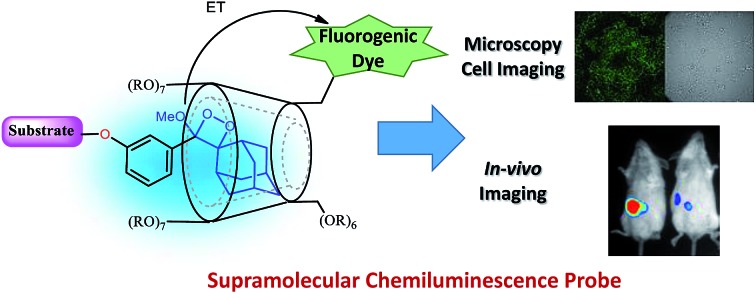
Chemiluminescence offers advantages over fluorescence for bioimaging, since an external light source is unnecessary with chemiluminescent agents.

## Introduction

Non-invasive acquisition of biomedical images at tissue and cellular levels is considered as a major challenge.^1^,^2^ A common bioimaging modality used today is based on optical fluorescence.^3^ Fluorescent imaging techniques offer high spatial and temporal resolution compared to magnetic resonance imaging (MRI), computed tomography (CT), and ultrasound imaging (USI).^4^,^5^ Moreover, fluorescence imaging devices are relatively inexpensive, easy to use, portable, and adaptable for detection and monitoring of various biological processes.^6^–^8^ However, an external light source is needed, and the signal-to-noise ratio in such measurements is often limited due to light interference resulting from excitation and autofluorescence of biological systems.^9^–^11^ Therefore, there is an obvious need for alternative optical bioimaging modalities that overcome these obstacles.

Bioimaging using a chemiluminescence modality offers attractive advantages over fluorescence imaging, mainly due to the fact that an external light source is unnecessary with chemiluminescent agents.^12^ Further, chemiluminescent assays are among the most sensitive methods for determination of enzymatic activity and analyte concentrations.^13^–^15^ Most known chemiluminescence probes emit light following a reaction with an oxidizing agent. Such probes usually undergo oxidation to form unstable strained peroxides that rapidly decompose to generate excited state intermediates that decay to the ground state through emission of light. This oxidation-based mechanism is widely utilized for activation of common chemiluminescent substrates such as luminol and oxalate esters.^16^

**Fig. 1:**
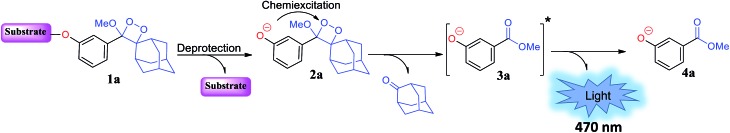
Molecular structure and activation pathway of triggerable 1,2-dioxetane.

In 1987, in pioneering work, the Schaap group reported a non-oxidative pathway for chemiluminescence activation based on triggerable phenoxy 1,2-dioxetanes.^17^–^19^ The general chemiexcitation pathway of phenoxy-dioxetane-based chemiluminescent probes is presented in Fig. 1. Dioxetane **1a** is equipped with an enzyme- or analyte-responsive substrate. Upon selective removal of the protecting group by an enzyme or an analyte of interest, phenolate-dioxetane species **2a** is released. The latter decomposes through a chemiexcitation process known as chemically initiated electron exchange luminescence (CIEEL) to produce benzoate ester **3a** in its electronically excited state. The decay of benzoate ester **3a** to its ground state **4a** is accompanied by emission of a blue photon. This class of 1,2-dioxetanes displays very high and efficient light emission in aprotic solvents such as dimethyl sulfoxide and acetonitrile.^17^

Under aqueous conditions, Schaap's dioxetanes suffer from major drawbacks of low solubility and low chemiluminescence quantum yield.^20^ The light emission efficiency is significantly decreased in aqueous solution relative to aprotic organic solvents as a result of non-radiative energy transfer that occurs upon interaction with surrounding water molecules.

This is likely due to the quenching effect of singlet excited anionic benzoate **3a** caused by phenomena such as dipole–dipole interactions, proton transfer, and hydrogen bonding between excited emitter **3a** and water molecules. The low quantum yield of the chemiluminescence process in aqueous medium was improved to some extent by Schaap, Matsumoto, and others through the addition of a surfactant or by tethering a fluorescent dye to the 1,2-dioxetane molecule.^21^–^25^

We have also explored new approaches to enhance the light emission of phenoxy-dioxetanes in water. A new methodology that significantly improved the emission ability of benzoate ester **3a** under aqueous conditions, simply by installing an acceptor substituent at the *ortho* position of the phenoxy 1,2-dioxetane, was recently reported.^26^ This structural motif afforded remarkable emission efficiency with significant chemiluminescence enhancement under physiological conditions. Our novel 1,2-dioxetane derivatives have also been demonstrated to be very efficient for *in vitro* and *in vivo* bioimaging.^27^–^32^ In addition, our group has developed a modular practical synthetic route for dioxetane-fluorophore conjugates. We prepared two chemiluminescent dioxetane probes tethered to fluorescein or quinone-cyanine (QCy) dyes.^33^ The conjugates exhibited enhanced light emission efficiency under aqueous conditions compared to the parent probe.

The chemiluminescence light efficiency of Schaap's dioxetanes under aqueous conditions can also be improved through a supramolecular interaction with a host molecule that offers a hydrophobic protective environment.^34^ Host molecules such as cyclodextrins (CDs) can form an inclusion complex with the hydrophobic probe and stabilize the intermediate formed during the chemiexcitation reaction. Here, we report a new host–guest inclusion complex between triggerable phenoxy-1,2-dioxetane and trimethylated-β-cyclodextrin (**TMCD**) that is suitable for use under physiological conditions for *in vitro* and *in vivo* imaging.

## Results

### Chemiluminescence enhancement effect obtained by complexation of adamantyl-dioxetane and CDs

CDs are cyclic oligosaccharides formed by monomers of d-glucopyranose bound together by means of α-1,4-glucosidic linkages that are closed into a ring to form a hollow truncated cone structure.^35^ They are classified based on the number of linked monomers: α-CDs consist of six units of glucose and β-CDs and γ-CDs consist of seven and eight glucose units, respectively.^36^ In a three-dimensional structure, the hydroxyl groups are on the outer edges, and the internal cavity contains only hydrogen atoms and oxygen bridges. Thus, CDs have an external hydrophilic surface and a hydrophobic central cavity. The central cavity of CDs (in particular that of β-CDs) can encapsulate hydrophobic molecules and, as a result, increase the solubility of the guest in water.^37^ CDs have been widely used in drug delivery, nucleic acid transfer, chiral separation of basic drugs, solubilization of lipophilic drugs, and molecular imaging.^38^–^44^

**Fig. 2:**
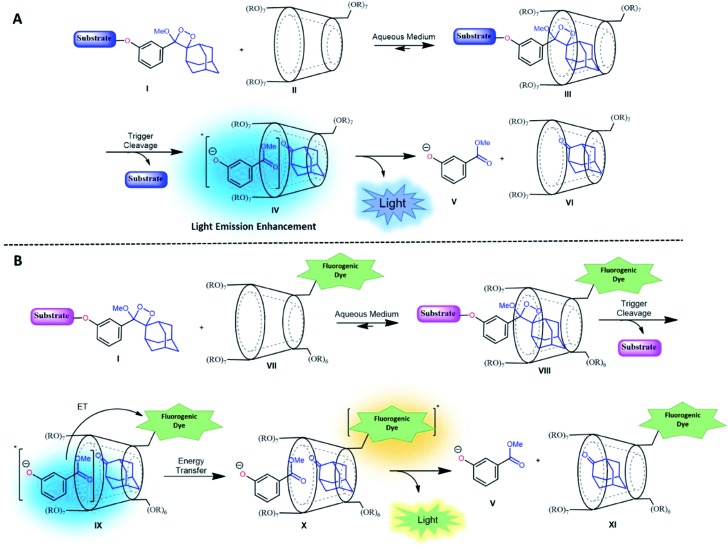
(A) Encapsulation of triggerable dioxetane **I** with β-CD derivatives. (B) Pathway of energy transfer of the probe in the presence of CDs tethered with a fluorophore.

It is well-known that adamantane guest molecules form highly stable complexes with β-CDs.^40^ The measured host–guest binding constant for adamantane with CDs is among the highest known for the formation of inclusion complexes because of the perfect fit of the adamantyl residue inside the β-CD cavity.^45^–^47^ As Schaap's dioxetane (**I**) contains an adamantyl moiety (Fig. 2A),^48^–^50^ we reasoned that CDs could be used to encapsulate 1,2-dioxetane probes. The β-CD-adamantane encapsulation process leads to formation of stable complex **III**. The CD cavity reduces water-induced quenching by providing a hydrophobic environment for the 1,2-dioxetane. Activation of the phenoxy 1,2-dioxetane generates excited state intermediate **IV**, and its decay to the ground state should be accompanied by enhanced light emission (Fig. 2A). To further enhance and to modulate the color of the chemiluminescence light emission of dioxetane **I**, the β-CD could covalently be attached to a fluorogenic dye as an energy transfer acceptor. In this way, the donor–acceptor space can be kept within the critical Foster distance by the host–guest complex. Energy transfer should take place efficiently from the donor (Schaap's dioxetane **I**) to β-CD-fluorogenic dye acceptor **VII** (Fig. 2B). Such energy transfer is expected to further enhance the light intensity under aqueous conditions with a red-shifted wavelength.^51^–^55^

**Fig. 3:**
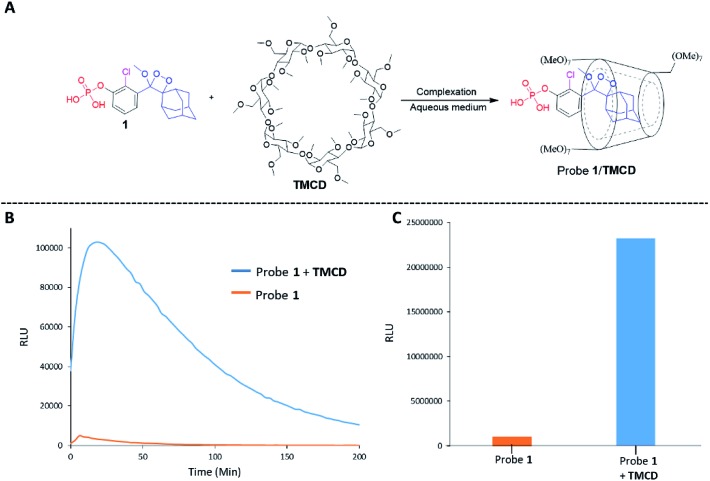
(A) Chemical structures of probe **1** and **TMCD**. (B) Chemiluminescence kinetic profile and (C) total photon counts of probe **1** [0.5 mM] and **TMCD** [9 mM] in Tris buffer, pH 9 (1% DMSO) in the presence of 1 unit per mL alkaline phosphatase.

Alkaline-phosphatase (AP) probe **1** (Fig. 3A) was chosen to evaluate the CD-encapsulation effect on the chemiluminescence emission in water. Numerous α-CD, β-CD, and γ-CD derivatives with various substituents on the hydroxyl groups were screened for their abilities to enhance the light emission of probe **1** in the presence of AP under aqueous conditions (Tris buffer, pH 9.0). The largest light emission enhancement effect was obtained with trimethylated-β-cyclodextrin (**TMCD**) as a host (Fig. 3A). This result could be explained through a structural circumstance, in which the secondary alcohols of the CD are located in close proximity to the adamantyl-dioxetane moiety encapsulation position. Such proximity could form hydrogen bond interactions with the dioxetane and its excited state intermediate.

These interactions lead to poor emission efficiency of Schaap's dioxetanes through a quenching effect. When the secondary alcohols are methylated, such a hydrogen bond quenching effect is avoided. The chemiluminescence light emission profile of probe **1** upon activation with AP was measured in the absence and presence of **TMCD** (Fig. 3B). The kinetic profile in the presence of **TMCD** was typical of a chemiluminescent probe with an initial signal increase to a maximum, in this case within 30 minutes, followed by a slow decrease to zero. **TMCD** significantly enhanced chemiluminescence with total photon counts emitted by probe **1** in the presence of **TMCD** being about 60-fold higher than those emitted by probe **1** in the absence of **TMCD** (Fig. 3C).

### Structural characterization and determination of the association constant between phenoxy-adamantyl-1,2-dioxetane and the TMCD host

**Fig. 4:**
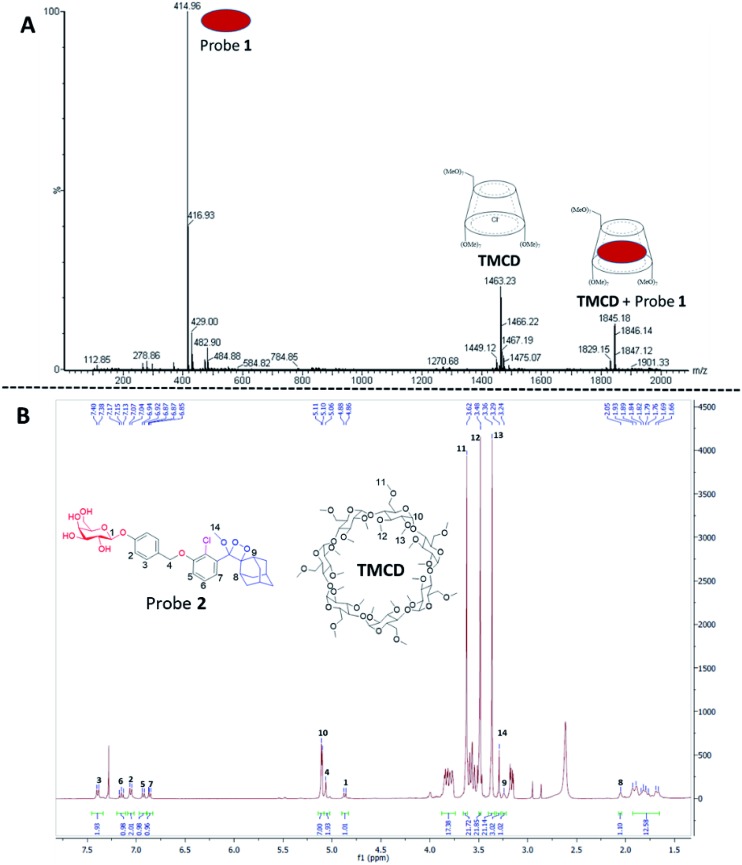
(A) Negative ESI spectra of an aqueous solution of probe **1** and **TMCD** prepared in a 1 : 1 molar ratio. (B) ^1^H-NMR spectrum of the crystalline supramolecular complex of probe **2** with **TMCD**. The crystallization experiment was carried out in H_2_O : DMF (95 : 5) with probe **2** and **TMCD** mixed at an initial molar ratio of 1 : 5.

The formation of a non-covalent inclusion complex between the **TMCD** host and alkaline phosphatase 1,2-dioxetane probe **1** in aqueous solution was validated by electrospray ionization mass spectrometry (ESI-MS).^56^,^57^ Fig. 4A shows the negative ion ESI spectrum acquired from an aqueous solution of **TMCD** and probe **1** in a molar ratio of 1 : 1. Three species are observed with *m*/*z* values of 414.96, 1463.23, and 1845.18, which adequately fit to [probe **1**]^–^, [**TMCD** + Cl]^–^, and the 1 : 1 complex [**TMCD** + probe **1**]^–^, respectively. This result indicates that there is a strong gas-phase interaction between the adamantyl moiety of probe **1** and the internal cavity of the CD derivative.

To provide additional evidence for the supramolecular structure obtained by the adamantyl probe and **TMCD**, we measured the ^1^H-NMR spectrum of supramolecular crystals of the complex of probe **2** with **TMCD** obtained after mixing the two components at a ratio of 1 : 5 in aqueous medium. Probe **2** is a phenoxy 1,2-dioxetane equipped with a β-galactosidase substrate. The NMR signals clearly correspond to a 1 : 1 complex of probe **2–TMCD** (Fig. 4B).

Next, ^31^P-NMR spectra were measured for solutions containing a fixed concentration of probe **1** (5 mM in D_2_O) and a variable concentration of the **TMCD** host (0.1–12.5 mM). Due to the chemical equilibrium of the complex formation, higher concentration of **TMCD** in the solution results in an increase in inclusion complex concentration. Consequently, the change of the ^31^P chemical shift provides a direct indication of the concentration of the complex formed between the guest and the host. The Benesi–Hildebrand equation was used for determination of the binding constant based on the following parameters: Δ*δ*, the chemical shift change of the phosphor of probe **1**; Δ*δ*_max_, the maximum chemical shift difference upon full complexation; [**TMCD**], the concentration of the host; and *K*, the equilibrium constant of the complexation.1
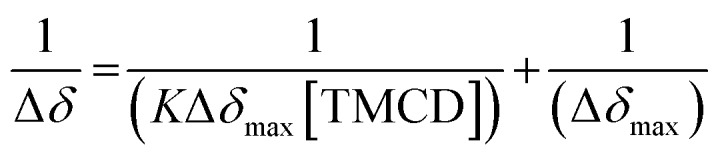



**Fig. 5:**
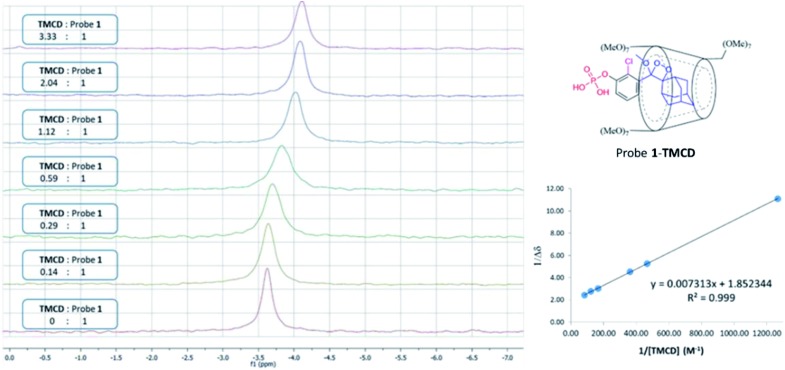
(Left) ^31^P-NMR titration experiment of **TMCD** and probe **1**. Conditions: 500 μL of D_2_O, 5 mg of probe **1** and up to 50 mg of **TMCD**. (Right) Correlation of 1/Δ*δ* as a function of probe **1/[TMCD]**, based on the Benesi–Hildebrand equation.

The ^31^P-NMR spectra of probe **1** upon incubation with various concentrations of **TMCD** are shown in Fig. 5. An excellent linear least–squares correlation was obtained by plotting 1/Δ*δ* as a function of **1/[TMCD]**. The plot clearly confirms a 1 : 1 intracavity complexation. The association constant *K*, calculated by determining the slope of the plot [1/(K*Δ*δ*_max_)] and the intercept with the vertical axis 1/Δ*δ*_max_, was 253 M^–1^. Comparison to previously reported examples^58^,^59^ clearly indicates that a relatively stable complex is formed between the phenoxy-adamantyl-1,2-dioxetane probe and the **TMCD** host (about 70% of the probe was complexed under the applied experimental conditions when using the indicated maximum concentrations of the probe and **TMCD**).

### *In vitro* and *in vivo* imaging applications of adamantyl dioxetane–cyclodextrin complexes

**Fig. 6:**
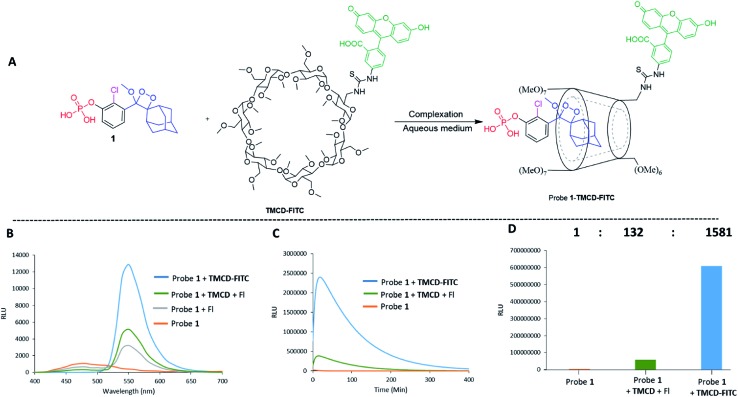
(A) Chemical structures of probe **1** and **TMCD-FITC**. (B) Chemiluminescence spectra of probe **1** [0.5 mM], probe **1** [0.5 mM] + **TMCD-FITC** [5 mM], probe **1** [0.5 mM] + Fl (fluorescein) [5 mM] and probe **1** [0.5 mM] + Fl [5 mM] + **TMCD** [5 mM] in Tris, pH 9 (1% DMSO), in the presence of 1 units per mL alkaline phosphatase. (C) Chemiluminescence kinetic profile and (D) total photon count of probe **1** [0.5 mM], probe **1** [0.5 mM] + **TMCD-FITC** [5 mM] and probe **1** [0.5 mM] + **TMCD** [5 mM] + Fl [5 mM] in Tris, pH 9 (1% DMSO), in the presence of 1 units per mL alkaline phosphatase.

Next, we wanted to evaluate if energy transfer in a complex of probe **1** with **TMCD** conjugated to a dye would further enhance the light emission intensity of the chemiluminescence reaction. Following the strategy presented in Fig. 2B, we synthesized a conjugate of a fluorescein dye with the **TMCD** host molecule (see ESI† for synthetic procedures). The chemical structure of the conjugate **TMCD-FITC** is shown in Fig. 6A. First, we measured the emission spectrum of probe **1** in comparison to that of a mixture of probe **1** and fluorescein, a mixture of probe **1**, **TMCD** and fluorescein (Fl) and a mixture of probe **1** and **TMCD-FITC** upon activation by AP. The spectra of the probe **1** and fluorescein mixture and probe **1**, **TMCD** and fluorescein mixture showed two emission maxima at wavelengths of 470 and 535 nm, corresponding to the direct chemiluminescence of probe **1** and to the emission of fluorescein resulting from partial energy transfer. The spectrum of the probe **1** and **TMCD-FITC** mixture exhibited a single maximum emission wavelength of 535 nm (Fig. 6B). This observation clearly indicates that energy transfer from the chemiluminescent probe to the dye occurs and thus validates the significance of the supramolecular effect obtained between the dioxetane probe and the dye facilitated by the **TMCD** host.

To examine the chemiluminescence enhancement of probe **1** complexed with **TMCD-FITC**, comparative experiments were carried out in the presence and absence of the **TMCD-FITC** conjugate. In both of the cases probe **1** exhibited a typical chemiluminescent kinetic profile with the signal increasing to a maximum, followed by a slow decrease to zero. In the presence of **TMCD-FITC**, probe **1** exhibited a remarkably strong chemiluminescence emission signal under aqueous conditions; however, probe **1** in the absence of the host produced an extremely weak emission signal (Fig. 6C). The total light emitted by the probe **1–TMCD-FITC** complex upon activation by AP was approximately 1500-fold higher than that emitted by the probe without the host (Fig. 6D). Incubation of free **TMCD**, fluorescein dye and probe **1** under similar conditions led to minor light emission enhancement.

The activation of Schaap's chemiluminescence probes is based on removal of a protecting group from the phenolic moiety. Therefore, different phenol protecting groups could be incorporated as triggering substrates for various analytes or enzymes.^60^,^61^ To demonstrate the modularity of our supramolecular enhancement approach, we synthesized three additional probes for detection of the enzymes β-galactosidase and penicillin-G-amidase (PGA) and the analyte hydrogen peroxide (see ESI† for synthetic procedures). The molecular structures of probes and their relative chemiluminescence light emissions are presented in Table 1. Probe **2** was equipped with a β-galactose group as a substrate for β-galactosidase, probe **3** with a phenylacetamide group as a substrate for PGA, and probe **4** with a phenylboronic ester as a substrate for hydrogen peroxide. Probes **2** and **3** were prepared with a chlorine substituent at the *ortho* position of the phenolic oxygen. The chlorine substituent was introduced in order to decrease the p*K*a of the released phenol obtained after the trigger removal. Such a p*K*a enables the chemiexcitation pathway of the dioxetane to occur under physiological conditions.

**Table 1:** Molecular structure and relative chemiluminescence parameters of probes **1–4** in the presence or absence of **TMCD** or **TMCD-FITC**

Chemiluminescence probe	Enzyme/analyte	Buffer	Relative CL emission (**TMCD**)	Relative CL emission (**TMCD-FITC**)
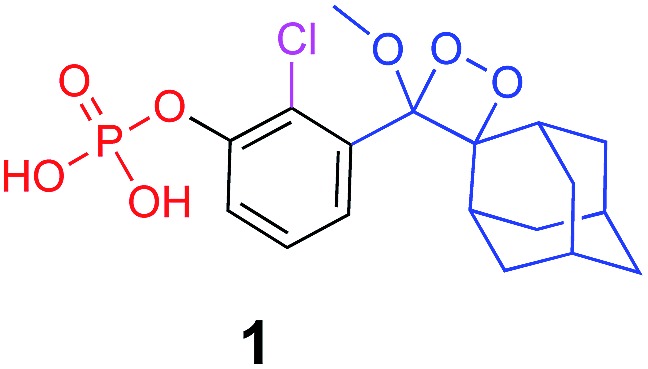	Alkaline phosphatase	Tris pH=9	60	1581
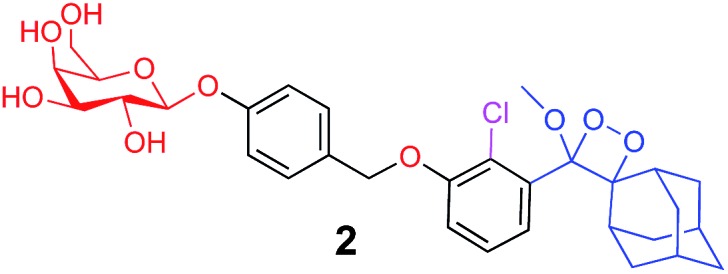	β-Galactosidase	PBS pH=7.4	15	410
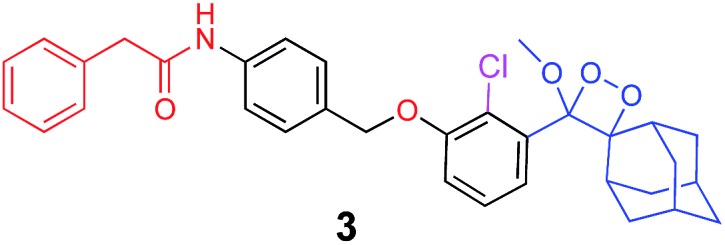	Penicillin-G-amidase	PBS pH=8.3	20	520
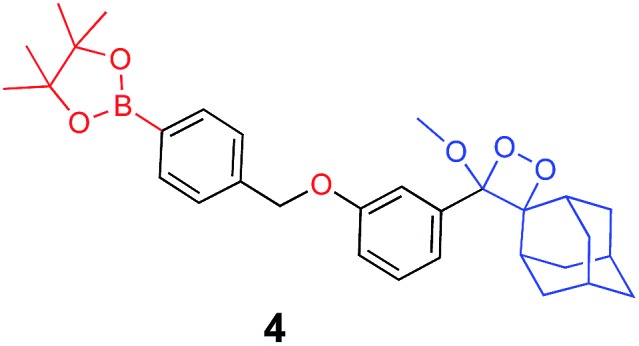	Hydrogen peroxide	Tris pH=10	47	1444

In comparative experiments, the chemiluminescence signals of the probes were measured in the presence and in the absence of **TMCD** or **TMCD-FITC** under the optimal pH conditions for the enzyme/analyte and the probes. The chemiluminescence emission of probe **2** upon activation with β-galactosidase in PBS, pH 7.4 was extremely weak. However, in the presence of **TMCD-FITC**, probe **2** exhibited a remarkably strong chemiluminescence emission signal with a 400-fold enhancement relative to the probe alone (15-fold for **TMCD**). In the presence of **TMCD-FITC**, probe **3** showed a similar signal enhancement, which is about 500-fold higher than that observed for probe **3** without the host upon the activation with the PGA enzyme in PBS, pH 8.3 (20-fold for **TMCD**). In the presence of **TMCD-FITC**, probe **4** exhibited the highest emission enhancement, almost 1500-fold higher than that of the probe without the host upon activation with hydrogen peroxide in Tris buffer, pH 10 (47-fold for **TMCD**).

**Fig. 7:**
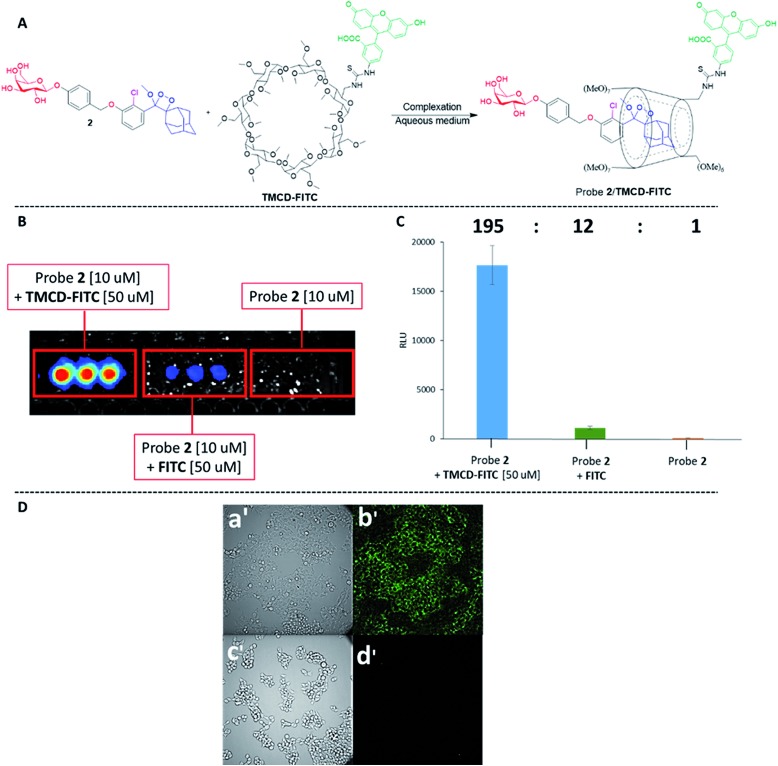
(A) Chemical structures of probe **2** and **TMCD-FITC**. (B) Chemiluminescence imaging and (C) quantification of signal intensities in HEK-293-LacZ cells incubated with the complex of probe **2** [10 μM] and **TMCD-FITC** [50 μM]. (D) (a′) Transmitted light image and (b′) chemiluminescence microscopy image of HEK-293-LacZ cells. (c′) Transmitted light image and (d′) chemiluminescence microscopy image of HEK-293-WT cells. Images were obtained following 20 min of incubation with a cell culture medium containing probe **2** [50 μM] and TMCD [50 μM]. Images were taken using an LV200 Olympus microscope using a 60 × objective and a 5 min exposure time.

The remarkable chemiluminescence enhancement obtained by complexation of **TMCD-FITC** with the phenoxy 1,2-dioxetane probes prompted us to examine whether the supramolecular complex could be used for live cell imaging. We first investigated the feasibility of the probe **2–TMCD-FITC** complex for imaging endogenous β-galactosidase activity in human embryonic kidney cells (HEK293) transfected with the *Lac-Z* gene. Probe **2–TMCD-FITC**, a mixture of probe **2** and **FITC**, and probe **2** were incubated with HEK-293-LacZ cells, and light emission was monitored (see ESI† for spectroscopic data). Remarkably, the probe **2–TMCD-FITC** complex generated an intense chemiluminescence signal when incubated with HEK-293-LacZ cells, whereas negligible signals were observed with the probe **2** and **FITC** mixture and with probe **2** alone. The quantified chemiluminescence signals are presented in Fig. 7B. The ratio between the signal intensity observed for the probe **2–TMCD-FITC** complex and that of probe **2** alone was about 195-fold. These results clearly demonstrate that the **TMCD-FITC-**phenoxy 1,2-dioxetane system serves as a chemiluminescence probe that can be used to image β-galactosidase activity in living cells.

We next evaluated the ability of our probe system to image HEK-293-LacZ cells using an LV200 microscope. HEK-293-LacZ and HEK293 cells that do not express β-galactosidase (HEK-293-WT) were incubated with the probe **2–TMCD-FITC** complex and then imaged using an LV200 microscope (Fig. 7D). The obtained results clearly show that the supramolecular complex formed by probe **2** and **TMCD-FITC** was able to produce chemiluminescence images of the HEK-293-LacZ cells (Fig. 7D(b′). No chemiluminescence signal was observed from HEK-293-WT cells (Fig. 7D(d′)). Although the recorded image quality is rather low, this is the first successful attempt to obtain chemiluminescence images using a supramolecular complex of phenoxy 1,2-dioxetanes.

**Fig. 8:**
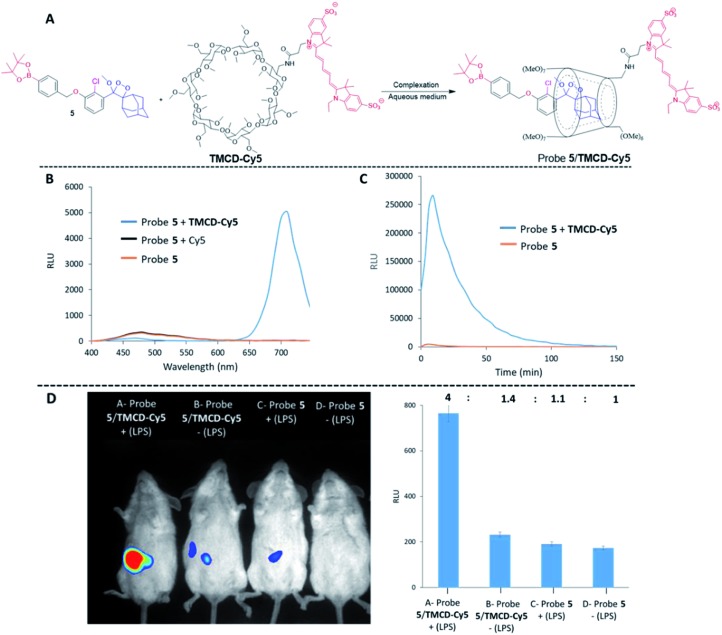
(A) Chemical structures of probe **5** and **TMCD–Cy5**. (B) Chemiluminescence spectra and (C) kinetic profiles of probe **5** [100 μM] and **TMCD–Cy5** [300 μM] in PBS, pH 7.4 (1% DMSO). (D) (Left) representative *in vivo* images of endogenous hydrogen peroxide in the peritoneal cavity of mice during an LPS-induced inflammatory response using probe **5** and **TMCD–Cy5**. The images of mice were recorded on a BioSpace Lab PhotonIMAGER. For the mice in group A, 1 mL of 0.1 mg mL^–1^ LPS was injected into the peritoneal cavity, followed 4 h later by an i.p. injection of probe **5** [100 μM] and **TMCD–Cy5** [300 μM] in 100 μL PBS, pH 7.4. For group B, 1 mL of PBS, pH 7.4 was injected i.p., followed 4 h later by an i.p. injection of probe **5** [100 μM] and **TMCD–Cy5** [300 μM] in 100 μL PBS, pH 7.4. For group C, 1 mL of 0.1 mg mL^–1^ LPS was injected i.p., followed 4 h later by an i.p. injection of probe **5** [100 μM] in 100 μL PBS, pH 7.4. For group D, 1 mL of PBS, pH 7.4 was injected i.p., followed 4 h later by an i.p. injection of probe **5** [100 μM] in 100 μL PBS, pH 7.4. (Right) quantification of signal intensities measured from each group of mice.

Finally, we evaluated the use of the supramolecular phenoxy-dioxetanes as *in vivo* chemiluminescence probes. It was essential to synthesize a host system that was equipped with a near-infrared (NIR) fluorescent dye for the *in vivo* application. The NIR region is useful for *in vivo* imaging, since such wavelengths better penetrate and are less scattered by living tissues than UV wavelengths. Using the strategy described in Fig. 2B, we synthesized the **TMCD–Cy5** host conjugate by tethering **TMCD** with the **Cy5** dye (see ESI† for synthetic procedures). Probe **5** can be used for imaging of reactive oxygen species (ROS) as it is equipped with a boronate trigger that can be activated with hydrogen peroxide. The chemical structure of probe **5** and the **TMCD–Cy5** conjugate and the chemiluminescence enhancement properties upon reaction with hydrogen peroxide are shown in Fig. 8A.

No energy transfer was observed when a mixture of probe **5** was activated by hydrogen peroxide in solution in the presence of** Cy5**. However, the activation of probe **5** in the presence of **TMCD–Cy5** resulted in almost complete energy transfer with light emission at a wavelength of 703 nm (Fig. 8B). The kinetic profiles of probe **5** with and without **TMCD–Cy5** were measured in PBS, pH 7.4 in the presence of hydrogen peroxide. As shown in Fig. 8C, the signal produced by probe **5** complexed with **TMCD–Cy5** was about 100-fold higher than that of probe **5** alone.

To demonstrate the ability of the chemiluminescence probe **5**-NIR **TMCD–Cy5** guest–host complex to serve as an imaging tool for *in vivo* use, we sought to image the biologically relevant ROS hydrogen peroxide.^62^ Since the overproduction of hydrogen peroxide *in vivo* is associated with the development of numerous inflammatory diseases, we investigated the ability of probe **5–TMCD–Cy5** to image endogenously produced hydrogen peroxide in a mouse model. Acute inflammation was induced by lipopolysaccharide (LPS). Mice were injected intraperitoneally (i.p.) with LPS (1 mL of 0.1 mg mL^–1^), followed 4 hours later by an additional i.p. injection of 100 μL of probe **5** [100 μM] and **TMCD–Cy5** [300 μM]. Imaging was performed using a non-invasive BioSpace Lab PhotonIMAGER system. Fig. 8D shows that a significantly greater intensity of the NIR light emission signal was observed from LPS-treated mice injected with probe **5–TMCD–Cy5** (group A) compared with non-LPS-treated mice injected with the complex (group B). The slight light emission signal observed in non-LPS-treated mice is attributed to basal levels of hydrogen peroxide produced by living animals. In mice treated with LPS and in control untreated mice, probe **5** alone resulted in a significantly lower and more non-selective light emission signal than did the complex (groups C and D). The signal-to-noise ratio of the NIR light emission intensity observed from probe **5–TMCD-Cy5** in mice treated with LPS was about 3-fold higher than that of the non-LPS-treated mice.

## Discussion

Currently, chemiluminescence signal enhancement is gaining increased attention. As mentioned above, our group recently reported the discovery of a new molecular methodology to significantly improve the light-emission efficiency of 1,2-dioxetane probes simply by installing various acrylate substituents at the *ortho* position of the phenoxy 1,2-dioxetane. The chemiluminescence light emission of the acrylate-substituted probes was up to 3000 fold higher than that of the known original adamantylidene–dioxetanes. In the present study, supramolecular complexation of the original 1,2-dioxetanes with cyclodextrin led to an increase of up to 1600 fold in chemiluminescence efficiency. Such a supramolecular approach possesses three key advantages: (i) there is no need for structural modification of the original Schaap's 1,2-dioxetane probe, (ii) complexation with fluorogenic dye-tethered cyclodextrin allows easy color modulation and red-shifting of the emitted light, and (iii) improved water solubility and chemical stability of the probe.

In commercial chemiluminescent immunoassays, a surfactant and a fluorescent dye are usually added together with adamantylidene–dioxetane probes. The surfactant reduces water-induced quenching by providing a hydrophobic environment for the excited chemiluminescent probe. The surfactant-fluorescent dye component can enhance the light emission of the dioxetane probe under aqueous conditions by 400-fold. However, since the surfactant mode of action relies on formation of micelles, its functional concentration is relatively high (above the critical micelle concentration, CMC). As micellar structures are not maintained when animals are treated systemically, the surfactant adduct approach is not practical for *in vivo* and cell applications.

## Conclusions

In summary, we have demonstrated the first molecular encapsulation of chemiluminescence phenoxy-adamantyl-1,2-dioxetane probes with trimethyl β-cyclodextrin. MS and NMR spectra provided clear proof for the formation of a stable 1 : 1 host–guest complex. The measured association constant of this host–guest system (253 M^–1^) indicates the formation of a stable inclusion complex between phenoxy-adamantyl-1,2-dioxetanes and the trimethyl β-cyclodextrin. This inclusion complex was able to amplify the light emission intensity by 60-fold under physiological conditions. Complexation of the adamantyl-dioxetane with fluorogenic dye-tethered cyclodextrin resulted in light emission through energy transfer to a longer wavelength, which corresponds to the fluorescent emission of the conjugated dye. Remarkably, the light emission intensity of such inclusion complexes was up to 1500-fold higher than that of the adamantyl-dioxetane guest alone.

Overall, these supramolecular complexes were shown to serve as efficient chemiluminescence probes for imaging of enzymatic activity and bio-analytes *in vitro* and *in vivo*. In addition, we demonstrated the first microscopy cell images obtained using chemiluminescence supramolecular dioxetane probes. We anticipate that the described chemiluminescence supramolecular dioxetane probes would find further use in various biological applications.

## Ethics statement

All animal procedures were performed in compliance with Tel Aviv University, Sackler School of Medicine guidelines and protocols approved by the Institutional Animal Care and Use Committee.

## Conflicts of interest

There are no conflicts to declare.

## Supplementary Material

Supplementary information
