# Supplementary material for: Light emission enhancement by supramolecular complexation of chemiluminescence probes designed for bioimaging
Source: Chem Sci. 2019 Jan 16;10(10):2945–55. doi: 10.1039/c8sc05174g (PMC6427943; doi:10.1039/c8sc05174g)
Supplement: Supplementary file 1 [file SC-010-C8SC05174G-s001.pdf]

Electronic Supplementary Material (ESI) for Chemical Science.
This journal is © The Royal Society of Chemistry 2019

Light Emission Enhancement by Supramolecular Complexation of Chemiluminescence Probes Designed for Bioimaging

Samer Gnaim[a], Anna Scomparin[b,c], Anat Eldar-Boock[b], Christoph R. Bauer[d], Ronit Satchi-Fainaro[b], and Doron Shabat[a]*

[a]School of Chemistry, Raymond and Beverly Sackler Faculty of Exact Sciences, Tel Aviv University, Tel Aviv 69978 Israel.

[b]Department of Physiology and Pharmacology, Sackler Faculty of Medicine, Tel Aviv University, Tel Aviv 69978 Israel.

[c] Department of Drug Science and Technology, University of Turin, Via P. Giuria 9, 10125 Turin, Italy.

[d]Bioimaging Center, University of Geneva, Geneva, Switzerland

Supporting Information

Table of Contents

1. General information S3
2. Synthetic procedures S4-S11
3. Chemiluminescence kinetic profiles of probes **1-4** with **TMCD** S12-S13
4. Chemiluminescence kinetic profiles of probes **1-4** with **TMCD-FITC** S13-S14
5. Chemiluminescence microscopy cell imaging data S15
6. Cell viability data S15
7. Proposed chemiluminescence enhancement mechanism S16
8. *In vitro* Experiments S18
9. *In vivo* Experiments S18-S19
10. MS Spectra S20-S21
11. ^{1}H-NMR Spectra S22-S25
12. References S26

General methods

Materials and instrumentations: All reactions requiring anhydrous conditions were performed under an argon atmosphere. All reactions were carried out at room temperature unless stated otherwise. Chemicals and solvents were either A.R. grade or purified by standard techniques. Thin layer chromatography (TLC): silica gel plates Merck 60 F254: compounds were visualized by irradiation with UV light. Flash chromatography (FC): silica gel Merck 60 (particle size 0.040-0.063 mm), eluent given in parentheses. Reverse-phase high pressure liquid chromatography (RP-HPLC): C18 5u, 250x4.6 mm, eluent given in parentheses. Preparative RP-HPLC: C18 5u, 250x21 mm, eluent given in parentheses. ^{1}H-NMR spectra were recorded using Bruker Avance operated at 400 MHz. ^{13}C-NMR spectra were recorded using Bruker Avance operated at 100 MHz. Chemical shifts were reported in ppm on the δ scale relative to a residual solvent ($CDCl_3$: δ = 7.26 for ^{1}H-NMR and 77.16 for ^{13}C-NMR, DMSO-d_6: δ = 2.50 for ^{1}H-NMR and 39.52 for ^{13}C-NMR). Mass spectra were measured on Waters Xevo TQD. Fluorescence and chemiluminescence were recorded on Molecular Devices Spectramax i3x. All reagents, including salts and solvents, were purchased from Sigma-Aldrich. All tissue culture materials were purchased from Biological Industries Ltd. (Kibbutz Beit Haemek, Israel), unless stated otherwise.

Abbreviations: AcOH - acetic acid, CAN - acetonitrile, DCM - dichloromethane, DMF - N,N'- dimethylformamide, EtOAc - ethyl acetate, Hex - hexanes, MeOH - methanol, TFA - trifluoroacetic acid, THF – tetrahydrofuran, DMAP – 4,4,dimethylaminopyridine, DBTL - Dibutyltin dilaurate.

Synthetic procedures

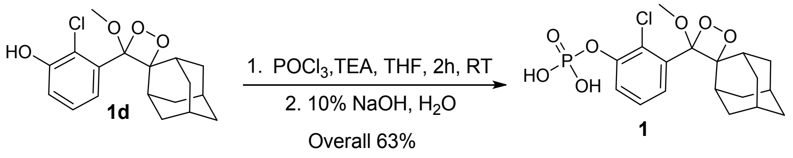

Compound **1:**

$POCl_3$ (2.5 mL, 26 mmol) and trimethylamine (7.3 mL, 52 mmol) were dissolved in 100 mL dry THF and cooled to 0 °C. Dioxetane **1d**[1] (800 mg, 2.6 mmol) was added dropwise over 60 minutes and the solution stirred at 0 °C for 60 minutes. After completion, the reaction mixture diluted with 10% NaOH (30 ml) and stirred for 30 min before quench with sat. NH_4Cl. The mixture diluted with EtOAc and the organic layer was separated, washed with brine, dried over Na_2SO_4 and evaporated under reduced pressure. The crude product was purified by preparative RP-HPLC to give compound **1** (681 mg, 63% yield). ^{1}H NMR (400 MHz, D_2O) δ 7.27-7.16 (m, 2H), 7.03 (d, J = 22.1 Hz, 1H), 3.22 (s, 3H), 2.99 (s, 1H), 1.96 (s, 1H), 1.69– 1.37 (m, 12H).; ^{13}C-NMR (100 MHz, D_2O) δ 156.96, 156.76, 138.84, 138.14, 133.42, 129.82, 129.82, 126.48, 121.93, 121.41, 117.14, 117.14, 115.17, 101.16, 100.77, 76.66, 74.80, 73.26, 71.81, 70.47, 62.26, 60.69, 38.66, 38.66, 38.66, 38.66, 37.93, 33.40, 33.40, 28.20, 28.20.; MS (ESI-) m/z calculated for $C_{18}H_{21}ClO_7P$ $[M - H]^-$ 415.3, found 415.6.

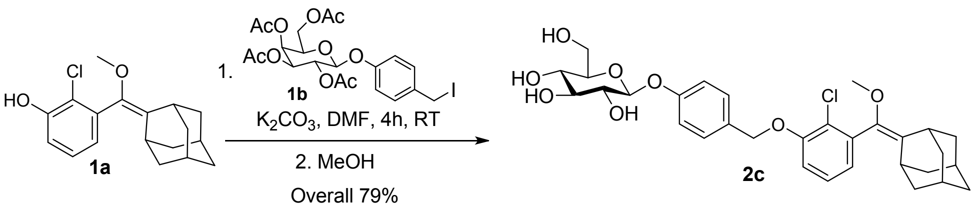

Compound **2c:**

Enol ether **1a**[1] (100 mg, 0.33 mmol) was dissolved in 5 mL dry DMF and cooled to 0 °C. K_2CO_3 (68 mg, 0.5 mmol) was added and the solution stirred at 0 °C for 10 minutes, before iodide **1b**[1] (372 mg, 0.66 mmol) was added. The reaction mixture stirred for 60 minutes at room temperature and monitored by TLC. After completion, the reaction mixture diluted with MeOH (10 ml) and stirred for 30 min before quench with sat. NH_4Cl. The mixture diluted with EtOAc and the organic layer was separated, washed with brine, dried over Na_2SO_4 and evaporated under reduced pressure. The crude product was purified by silica gel column chromatography to afford the corresponding product **2c** (149 mg, 79% yield). ^{1}H NMR (400 MHz, MeOD) δ 7.40 (d, J = 8.6 Hz, 1H), 7.25 – 7.17 (m, 1H), 7.10 (dd, J = 17.6, 8.4 Hz, 2H), 6.85 (d, J = 1.2 Hz, 1H), 5.09 (s, 2H), 4.94 – 4.84 (m, 5H), 3.90 (d, J = 3.2 Hz, 1H), 3.80 – 3.70 (m, 2H), 3.60 – 3.49 (m, 7H), 3.32 – 3.20 (m, 5H), 2.09 – 1.67 (m, 13H). ^{13}C-NMR (100 MHz, $CDCl_3$) δ 170.40, 170.29, 170.16, 169.45, 167.91, 156.78, 155.84, 141.49, 139.50, 137.45, 133.77, 133.35, 131.20, 129.34, 128.88, 123.52, 118.62, 117.07, 115.42, 99.1, 71.05, 70.84, 70.02, 68.63, 66.87, 63.38, 61.34, 57.61, 51.65, 39.21, 37.02, 32.58, 30.20, 28.17, 20.76, 20.68, 20.61 ppm; MS (ESI+) m/z calculated for $C_{31}H_{37}ClO_8Na$ $[M + Na]^+$ 595.3, found 595.1.

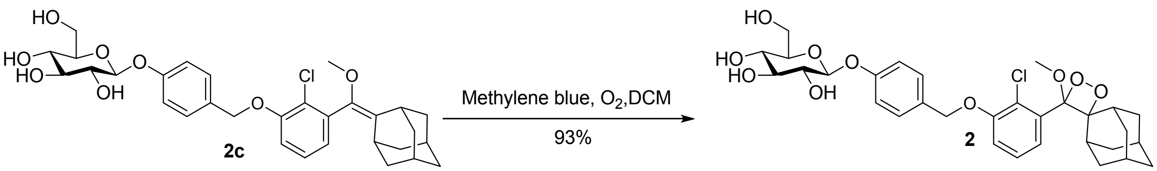

Compound **2**:

Enol ether **2c** (30 mg, 0.33 mmol) and catalytic amount of methylene blue were dissolved in 20 ml of DCM. Oxygen was bubbled through the solution while irradiating with yellow light. The reaction was monitored by RP-HPLC. After completion, the reaction mixture was concentrated by evaporation under reduced pressure. The crude product was purified by preparative RP-HPLC to give compound **2** (29 mg, 93 %). ^{1}H-NMR (400 MHz, $CDCl_3$) δ 7.67 (d, J = 17.8 Hz, 1H), 7.73-7.26 (m, 3H), 7.04–7.01 (m, 3H), 5.02 (s, 2H), 4.88– 4.86 (d, J = 20.1 Hz, 1H), 4.00 (s, 1H), 3.71 (s, 1H), 3.57 – 3.49 (m, 5H), 3.19 (s, 3H), 2.87 (s, 1H), 2.13 (s, 1H), 1.88-1.70 (m, 12H). ^{13}C-NMR (100 MHz, $CDCl_3$) δ 156.96, 156.76, 138.84, 138.14, 133.42, 129.82, 129.82, 126.48, 121.93, 121.41, 117.14, 117.14, 115.17, 101.16, 100.77, 76.66, 74.80, 73.26, 71.81, 70.47, 62.26, 60.69, 38.66, 38.66, 38.66, 38.66, 37.93, 33.40, 33.40, 28.20, 28.20. MS (ESI+) m/z calculated for $C_{31}H_{38}ClO_{10}$ $[M + H]^+$, 597.3 found 597.2.

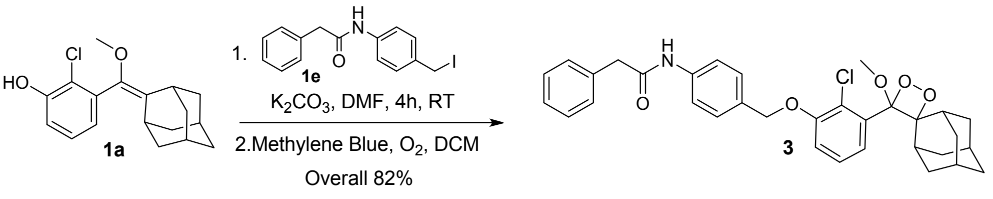

Compound **3:**

Enol ether **1a** (100 mg, 0.33 mmol) was dissolved in 5 mL dry DMF and cooled to 0 °C. K_2CO_3 (68 mg, 0.5 mmol) was added and the solution stirred at 0 °C for 10 minutes, before iodide **1b** (231 mg, 0.66 mmol) was added. The reaction mixture stirred for 60 minutes at

room temperature and monitored by TLC. After completion, the reaction mixture diluted with EtOAc and the organic layer was separated, washed with brine, dried over Na_2SO_4 and evaporated under reduced pressure. The crude product and catalytic amount of methylene blue were dissolved in 20 ml of DCM. Oxygen was bubbled through the solution while irradiating with yellow light. The reaction was monitored by RP-HPLC. After completion, the reaction mixture was concentrated by evaporation under reduced pressure. The crude product was purified by preparative RP-HPLC (gradient of ACN in water) to give probe **3** (146 mg, 82% yield). ^{1}H-NMR (400 MHz, $CDCl_3$) δ 7.44 – 7.34 (m, 10H), 7.33 – 7.28 (m, 2H), 5.19 (s, 2H), 3.75 (s, 2H), 3.18 (s, 3H), 3.01 (s, 1H), 2.22 (m, 1H), 2.15 – 1.51 (m, 13H); ^{13}C-NMR (100 MHz, $CDCl_3$) δ 172.47, 157.52, 139.22, 138.07, 134.78, 132.64, 129.45, 129.45, 129.18, 129.18, 128.42, 128.27, 128.27, 126.78, 126.56, 122.54, 120.77, 120.77, 117.86, 114.97, 91.94, 71.43, 52.09, 43.43, 37.93, 34.88, 34.88, 33.62, 33.62, 33.62, 33.62, 30.31, 30.31.ppm; MS (ESI+) *m*/*z* calculated for $C_{33}H_{34}ClNO_5Na$ $[M + Na]^+$ 582.7, found 583.1.

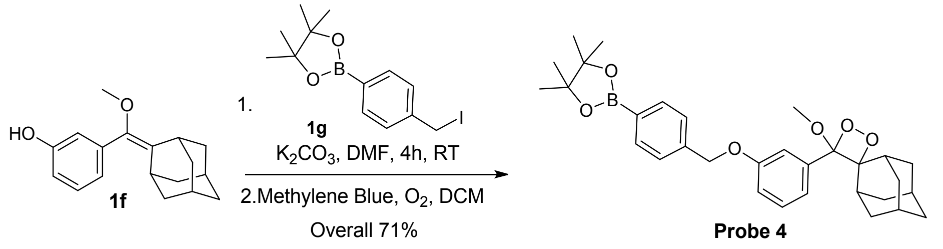

Compound **4:**

Enol ether **1f** (100 mg, 0.37 mmol) was dissolved in 5 mL dry DMF and cooled to 0 °C. K_2CO_3 (72 mg, 0.52 mmol) was added and the solution stirred at 0 °C for 10 minutes, before iodide **1g** (254 mg, 2.2 mmol) was added. The reaction mixture stirred for 4 hours at room temperature and monitored by TLC. After completion, the reaction mixture diluted

with EtOAc and the organic layer was separated, washed with brine, dried over Na_2SO_4 and evaporated under reduced pressure. The crude product and catalytic amount of methylene blue were dissolved in 20 ml of DCM. Oxygen was bubbled through the solution while irradiating with yellow light. The reaction was monitored by RP-HPLC. After completion, the reaction mixture was concentrated by evaporation under reduced pressure. The crude product was purified by preparative RP-HPLC (gradient of ACN in water) to give probe **4** (98 mg, 51% yield). ^{1}H-NMR (400 MHz, $CDCl_3$) δ 7.81(d, J = 16.0 Hz, 1H), 7.48 (s, 1H), 7.46-7.43 (m, 3H), 7.33-7.31(d, J = 8.4 Hz, 2H), 5.13 (s, 2H), 3.22 (s, 3H), 2.99 (s, 1H), 2.16-1.44 (m, 13H), 1.33(m, 12H); ^{13}C-NMR (100 MHz, $CDCl_3$) δ 160.33, 141.01, 139.27, 133.05, 133.05, 127.48, 127.10, 127.10, 121.37, 121.07, 120.83, 117.98, 116.30, 92.60, 87.49, 87.49, 70.84, 52.09, 37.93, 34.88, 34.88, 33.62, 33.62, 33.62, 33.62, 30.31, 30.31, 24.70, 24.70, 24.70, 24.70.; MS (ESI+) m/z calculated for $C_{31}H_{40}BO_6$ $[M + H]^+$ 519.3, found 519.6.

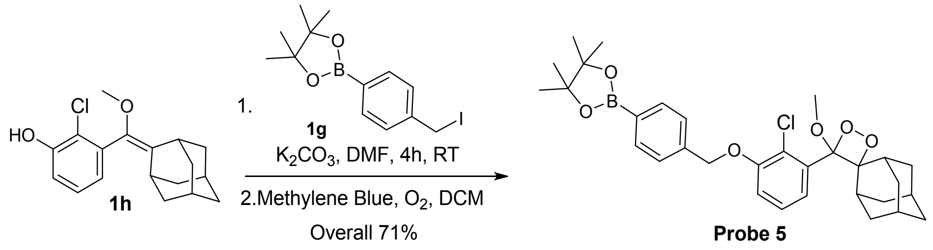

Compound **5:**

Enol ether **1a** (100 mg, 0.33 mmol) was dissolved in 5 mL dry DMF and cooled to 0 °C. K_2CO_3 (68 mg, 0.5 mmol) was added and the solution stirred at 0 °C for 10 minutes, before iodide **1g** (247 mg, 0.66 mmol) was added. The reaction mixture stirred for 4 hours at room temperature and monitored by TLC. After completion, the reaction mixture diluted with EtOAc and the organic layer was separated, washed with brine, dried over Na_2SO_4 and

evaporated under reduced pressure. The crude product and catalytic amount of methylene blue were dissolved in 20 ml of DCM. Oxygen was bubbled through the solution while irradiating with yellow light. The reaction was monitored by RP-HPLC. After completion, the reaction mixture was concentrated by evaporation under reduced pressure. The crude product was purified by preparative RP-HPLC (gradient of ACN in water) to give probe **5** (113 mg, 62% yield). ^{1}H-NMR (400 MHz, $CDCl_3$) δ 7.74(d, J = 16.0 Hz, 1H), 7.37 (d, J = 8.4 Hz, 2H), 7.17 (t, J = 8.4 Hz, 1H), 7.00 (d, J = 8.2 Hz, 1H), 6.83 (d, J = 16.0 Hz, 1H), 5.47-5.44 (m, 2H), 4.7 (s, 2H), 3.31-3.27 (m, 4H), 2.16 (s, 1H), 1.93-1.73 (m, 12H), 1.34 (s, 12H); ^{13}C-NMR (100 MHz, $CDCl_3$) δ 170.40, 170.29, 170.16, 169.45, 167.91, 156.78, 155.84, 141.49, 139.50, 137.45, 133.77, 133.35, 131.20, 129.34, 128.88, 123.52, 118.62, 117.07, 115.42, 99.1, 71.05, 70.84, 70.02, 68.63, 66.87, 63.38, 61.34, 57.61, 51.65, 39.21, 37.02, 32.58, 30.20, 28.17, 20.76, 20.68, 20.61 ppm; MS (ESI+) m/z calculated for $C_{31}H_{38}BClO_6Na$ $[M + Na]^+$ 575.3, found 575.3.

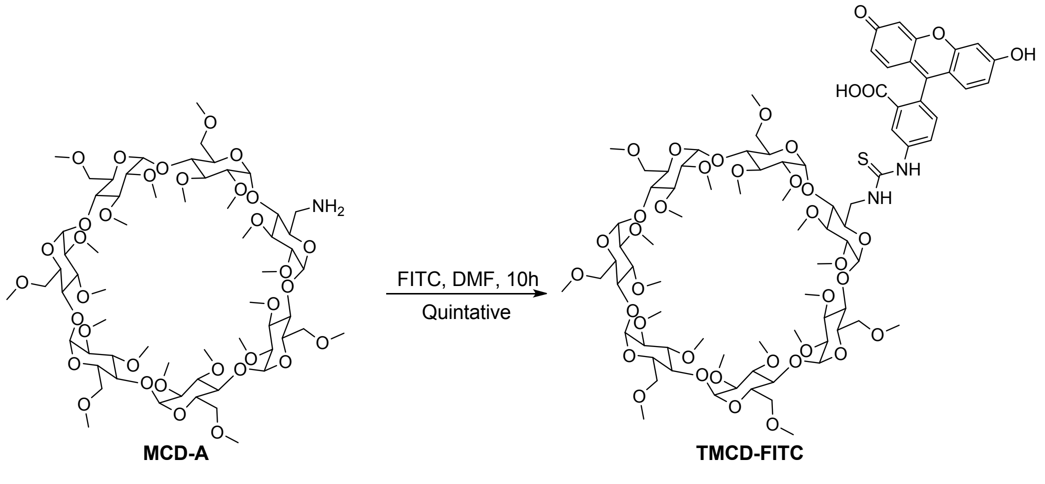

TMCD-FITC:

FITC (66 mg, 0.17 mmol) was added to a solution of **MCD-A** (200 mg, 0.14 mmol) in 3 mL DMF and stirred for 10h. After completion, the mixture diluted with water and purified by preparative RP-HPLC (gradient of ACN in water) to give **TMCD-FITC** (250 mg, 100%

yield). ^{1}H-NMR (400 MHz, CD_3OD) 10.02 (s, 2H), 8.07 (d, J = 13.2, 1H), 7.67-7.65 (m, 2H) 6.85 (d, J = 8.0, 1H), 6.62-6.54 (m, 6H), 5.41 (s, 1H), 5.30-5.35 (m, 6H), 3.86-3.96 (m, 16H), 3.87-3.92 (s, 21H), 3.75-3.83 (m, 21H); 3.76 (s, 18H), 3.69-3.79 (m, 18H), 3.65-3.73 (m, 9H). ^{13}C-NMR (100 MHz, CD_3OD) δ 187.05, 177.96, 168.69, 160.67, 156.02, 149.20, 146.47, 132.09, 130.90, 128.49, 127.62, 127.22, 125.06, 124.37, 121.37, 118.61, 118.12, 117.63, 110.93, 104.33, 103.09, 100.71, 100.71, 100.71, 100.71, 100.71, 100.71, 100.71, 83.71, 83.71, 83.71, 83.71, 83.71, 83.71, 83.71, 82.94, 82.94, 82.94, 82.94, 82.94, 82.94, 82.94, 79.03, 75.73, 75.73, 75.73, 75.73, 75.73, 75.73, 74.24, 72.43, 72.43, 72.43, 72.43, 72.43, 72.43, 71.82, 71.82, 71.82, 71.82, 71.82, 71.82, 58.84, 58.84, 58.84, 58.84, 58.84, 58.84, 58.84, 58.84, 58.84, 58.84, 58.84, 58.84, 58.84, 58.84, 58.84, 58.84, 58.70, 58.70, 58.70, 58.70, 58.70, 58.70, 58.70, 44.35. MS (ESI+) *m/z* calculated for $C_{83}H_{123}N_2O_{39}S$ $[M + H]^+$ 1803.9, found 1804.4.

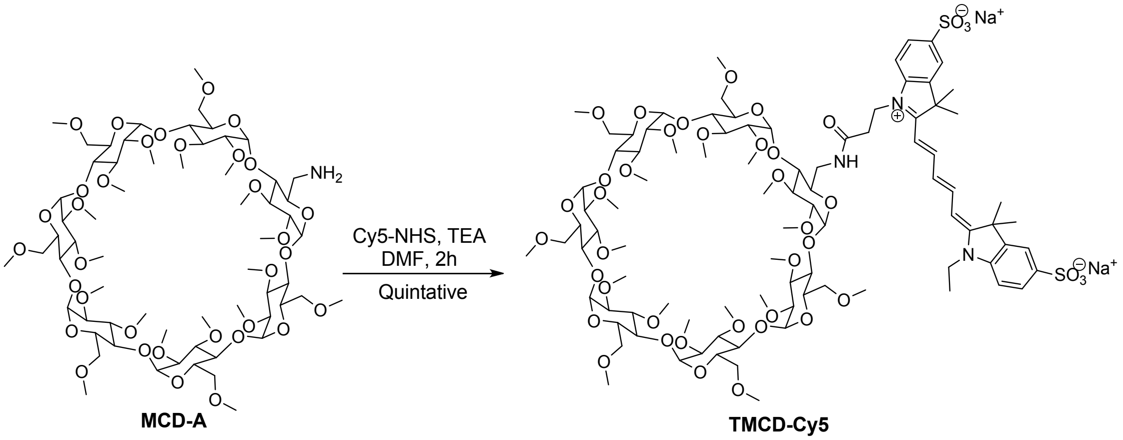

TMCD-Cy5:

Cy5-NHS (5.5 mg, 0.008 mmol) was added to a solution of **MCD-A** (10 mg, 0.007 mmol) and trimethylamine (2 μL, 0.014 mmol) in 3 mL DMF and stirred for 2h. After completion, the mixture diluted with water and purified by preparative RP-HPLC (gradient of ACN in

water) to give **TMCD-Cy5** (15 mg, 100% yield). ^{1}H-NMR (400 MHz, CD_3OD) 8.25 (t, *J*=13.2 Hz, 2H), 7.75-7.41 (m, 7H), 7.8 (s, 2 H), 7.65 (d, 2H, *J*=7.7 Hz), 7.3- 7.4 (m, 6H), 6.5 (t, 1 H, *J*=13 Hz), 6.2 (d, 2 H, *J*=13 Hz), 5.33 (s, 1H), 5.30-5.35 (m, 6H), 4.31 (m, 1H), 3.86-3.96 (m, 12H), 3.87-3.92 (m, 6H), 3.75-3.83 (m, 4H); 3.69-3.79 (m, 21H); 3.71 (s, 6H); 3.65-3.73 (m, 21H); 3.64-3.66 (m, 18H); 3.55-3.57 (m, 7H); 3.43-1.71 (s, 12H). ^{13}C-NMR (100 MHz, CD_3OD) δ 176.54, 171.22, 168.58, 153.22, 145.91, 142.25, 140.88, 140.01, 138.99, 138.20, 138.02, 131.50, 123.93, 123.52, 123.09, 123.02, 112.65, 109.39, 106.42, 100.71, 100.71, 100.71, 100.71, 100.71, 100.71, 100.71, 99.53, 83.71, 83.71, 83.71, 83.71, 83.71, 83.71, 83.71, 82.94, 82.94, 82.94, 82.94, 82.94, 82.94, 82.94, 79.03, 75.73, 75.73, 75.73, 75.73, 75.73, 75.73, 74.24, 72.43, 72.43, 72.43, 72.43, 72.43, 72.43, 71.82, 71.82, 71.82, 71.82, 71.82, 71.82, 58.84, 58.84, 58.84, 58.84, 58.84, 58.84, 58.84, 58.84, 58.84, 58.84, 58.84, 58.84, 58.84, 58.84, 58.70, 58.70, 58.70, 58.70, 58.70, 58.70, 50.18, 49.27, 45.34, 41.37, 41.09, 37.47, 28.27, 28.27, 26.14, 26.14, 13.65. MS (ESI+) *m*/2*z* calculated for $C_{92}H_{142}N_3Na_3O_{41}S_2$ $[M + Na]^{2+}/2$ 1047.2, found 1047.0.

Chemiluminescence kinetic profiles of probes 1-4 with TMCD:

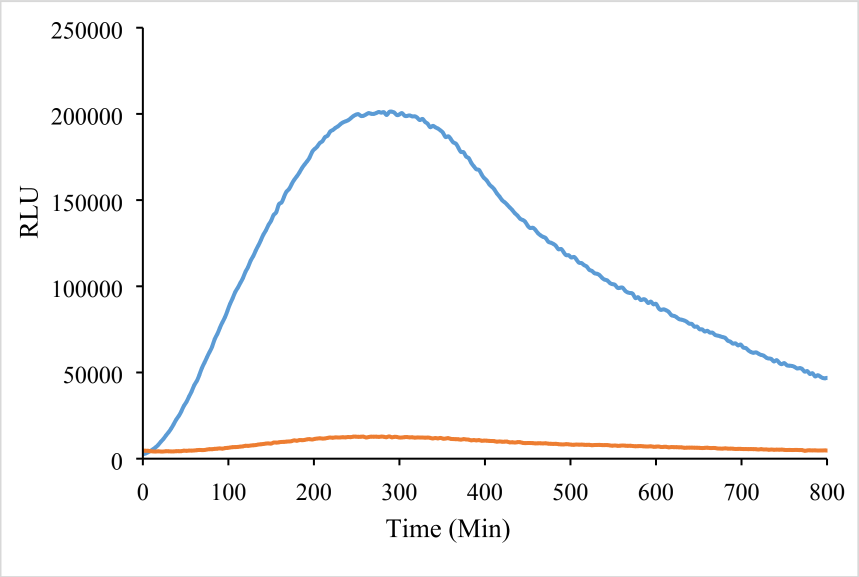

Figure S1: Chemiluminescence kinetic profiles of probe **2** [0.5 mM], TMCD [9 mM] and 1 EU of AP enzyme, in PBS 7.4 (1% DMSO), in the presence (blue) or absence (orange) of **TMCD**.

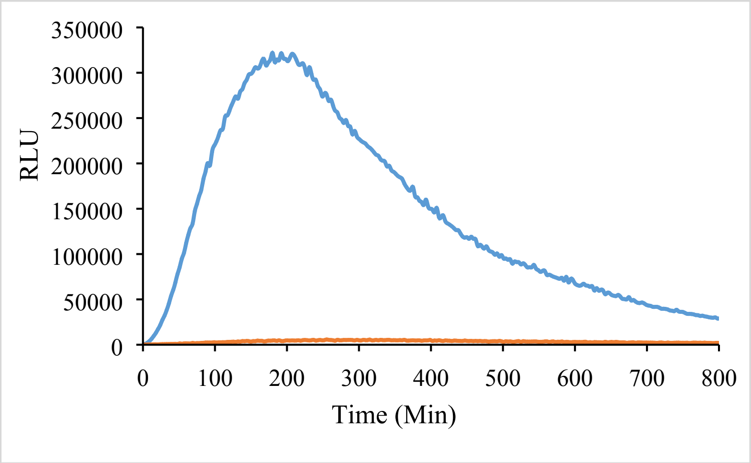

Figure S2: Chemiluminescence kinetic profile of probe **3** [0.5 mM], TMCD [9 mM] and 2 EU of PGA enzyme, in PBS 8.3 (1% DMSO), in the presence (blue) or absence (orange) of **TMCD**.

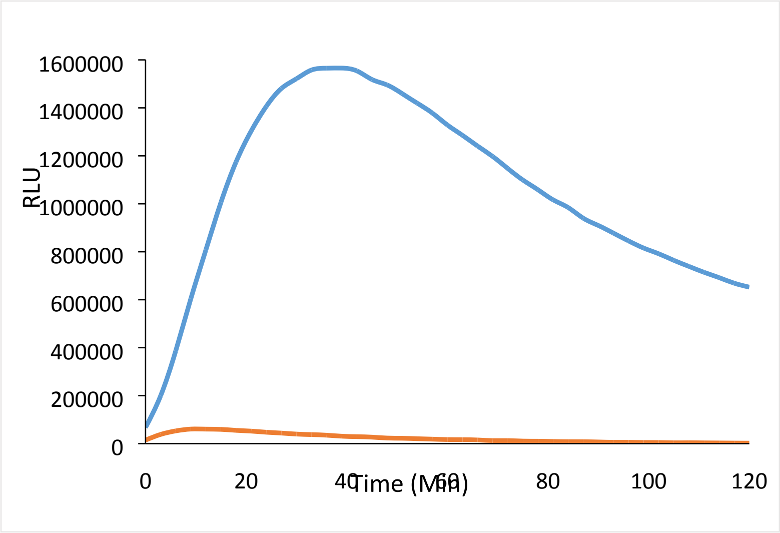

Figure S3: Chemiluminescence kinetic profile of probe **4** [0.5 mM], TMCD [9 mM] and H_2O_2 [0.5 mM], in Tris pH 10 (1% DMSO), in the presence (blue) or absence (orange) of **TMCD**.

<u>Chemiluminescence kinetic profiles of probes 1-4 with TMCD-FITC:</u>

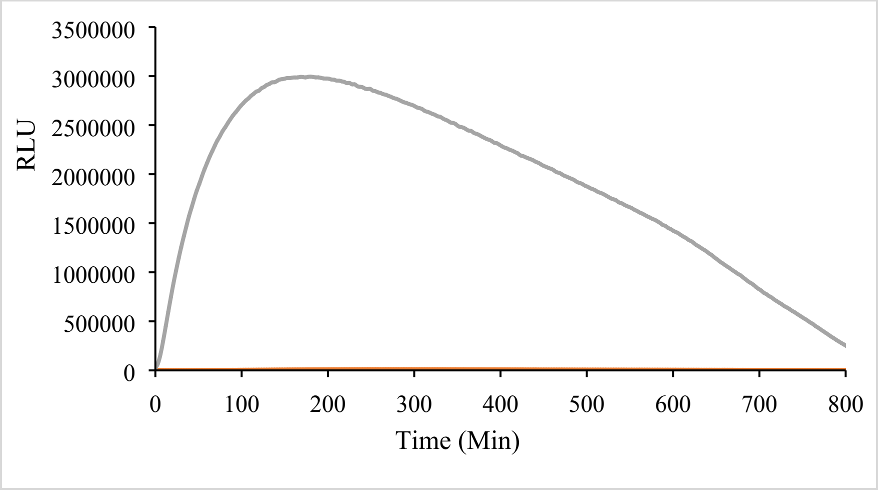

Figure S4: Chemiluminescence kinetic profile of probe **2** [0.5 mM], TMCD [5 mM] and 1 EU of AP enzyme, in PBS 7.4 (1% DMSO), in the presence (gray) or absence (orange) of **TMCD-FITC**.

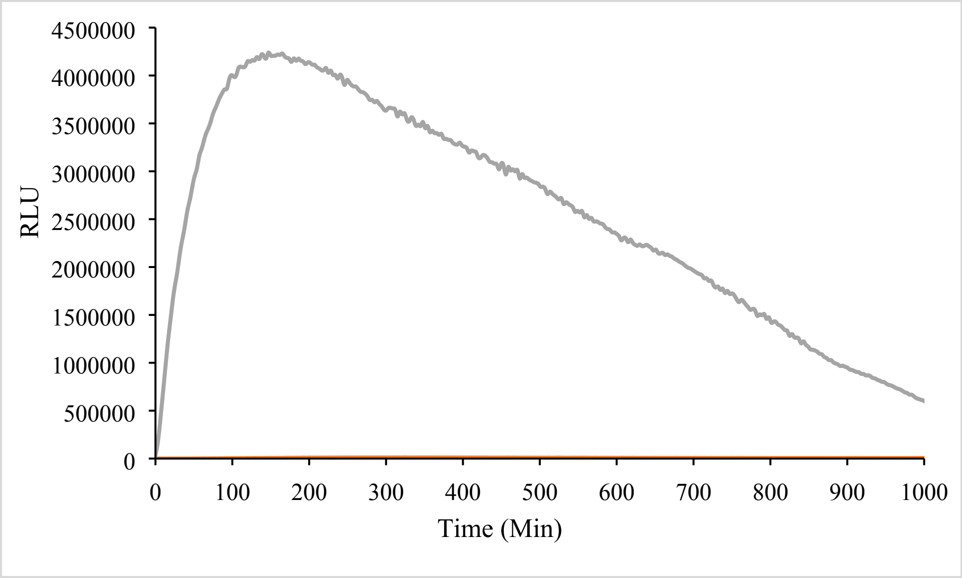

Figure S5: Chemiluminescence kinetic profile of probe **3** [0.5 mM], TMCD [5 mM] and 2 EU of PGA enzyme, in PBS 8.3 (1% DMSO), in the presence (gray) or absence (orange) of **TMCD-FITC**.

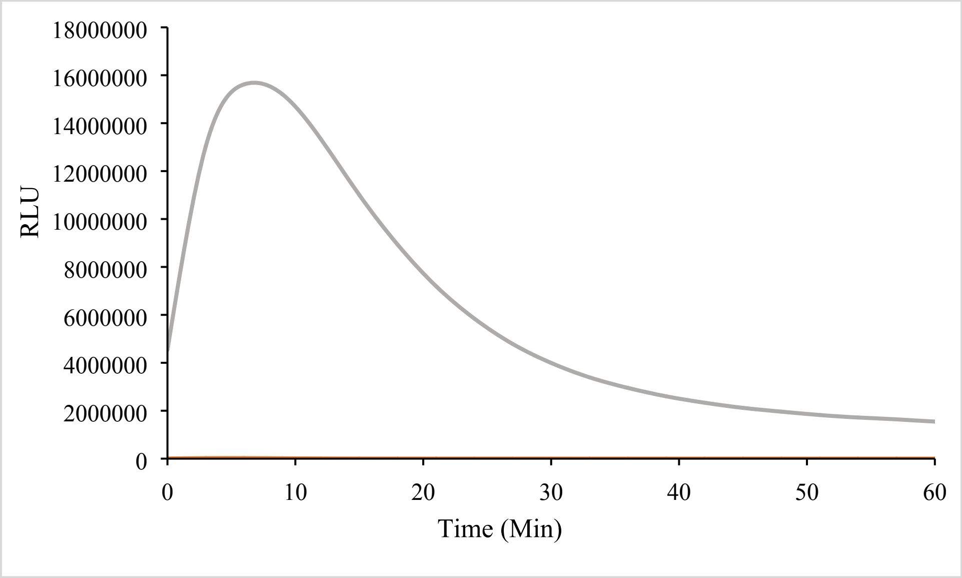

Figure S6: Chemiluminescence kinetic profile of probe **4** [0.5 mM], TMCD [9 mM] and H_2O_2 [0.5 mM], in Tris pH 10 (1% DMSO), in the presence (gray) or absence (orange) of **TMCD-FITC**.

Chemiluminescence microscopy cell imaging data

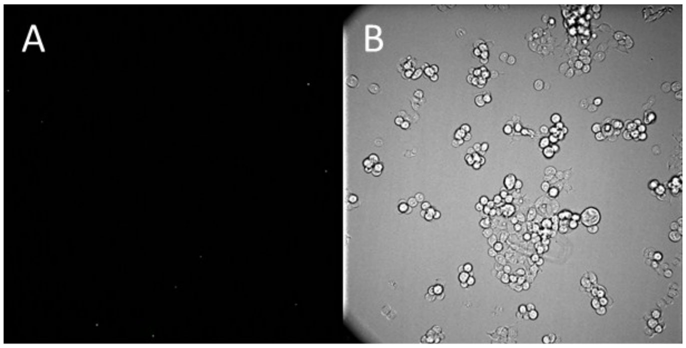

Figure S7: (A) Chemiluminescence microscopy image and (B) transmitted light image of HEK-293-LacZ cells. Images were obtained following 20 min incubation with cell culture medium containing probe **2** [50 μM]. Images were taken using the LV200 Olympus microscope using a 60× objective and 5 min exposure time.

Cell viability data

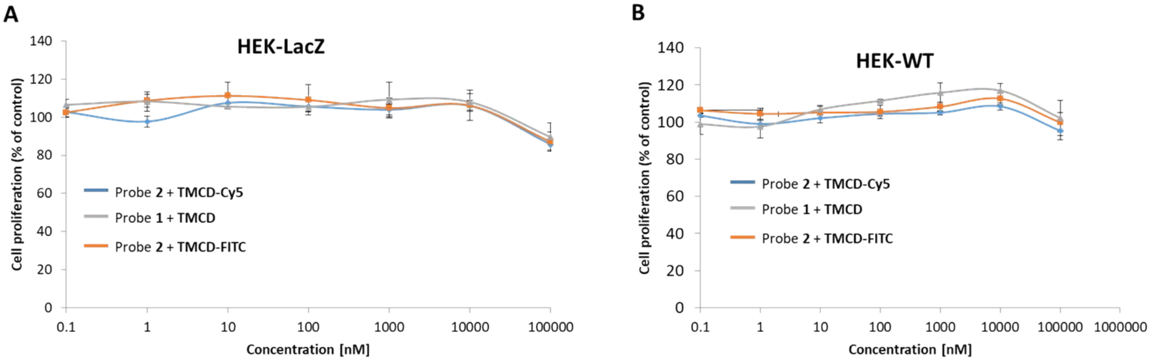

Figure S8: Cell viability experiment of the supramolecular complexes with A) HEK-LacZ cell line and, B) HEK-WT cell line.

Proposed chemiluminescence enhancement mechanism

To better understand the enhancement mechanism of the **TMCD** with Schaap's 1,2-dioxetane, we have examined the fluorescence enhancement exhibited for the emitter (benzoate) and **TMCD**. The direct chemiluminescence generated by emission of the corresponding dioxetane probe in water is directly affected from the fluorescence efficiency of the benzoate ester. As presented in Figure S10, the fluorescence of 3-hydroxybenozte (**3-HB**) was significantly higher after incubation with **TMCD** compared to that of the control experiment. The complexation with TMCD improved the fluorescence intense signal of **3-HB** with almost 13-folds. This result supports our proposed CL enhancement mechanism using supramolecular enhancer adducts. The chemiluminescence probe is encapsulated with **TMCD** to form a stable inclusion, then, activation of the probe led to the generation of the excited benzoate to emit an enhanced chemiluminescent light.

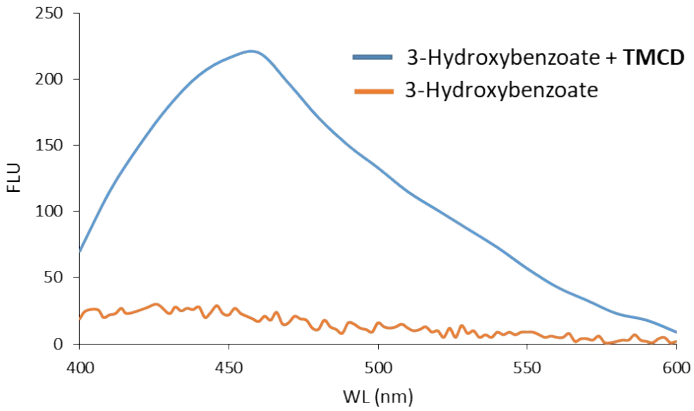

Figure S10: Fluorescence spectra of **3-HB** with and without **TMCD**.

Correlation between TMCD and TMCD-FITC Chemiluminescence Enhancement

In order to examine the complexation effect of **TMCD-FITC** compared to that of **TMCD**, we have plotted the relative chemiluminescence enhancement of **TMCD-FITC** as a

function of the relative enhancement achieved with **TMCD** for each probe (**1-4**). As shown in Figure S11, an excellent linear correlation was obtained with R^2 of 0.975. This result means that the complexation effect and strength of the CD derivatives is similar with probes **1-4**. Therefore the only difference is the energy transfer achieved with the **TMCD-FITC**, which leads to further 30-folds higher light emission.

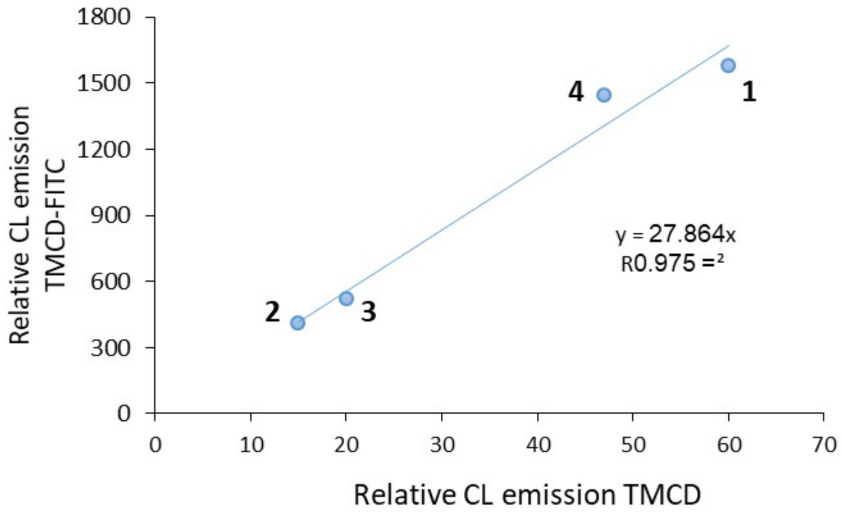

Figure S11: Correlation between the relative chemiluminescence enhancement using **TMCD** and **TMCD-FITC** with probes **1-4**.

The equilibrium constant calculation:

$$\frac{1}{\Delta\delta} = \frac{1}{(K \cdot \Delta\delta_{max} \cdot [TMCD])} + \frac{1}{(\Delta\delta_{max})}$$

$$\frac{1}{(K \cdot \Delta\delta_{max})} = 0.007313, \ \frac{1}{(\Delta\delta_{max})} = 1.852$$

$$K = 253\ 1/M$$

***In vitro* Experiments**

Cell Culture

HEK-293-WT cells were purchased from the American Type Culture Collection (ATCC Manassas, VA, USA). HEK-293-LacZ (β-Galactosidase) cell line was kindly provided by Christoph Ruediger Bauer (ETH, Zurich). All cell lines were cultured in DMEM growing media supplemented with 10% FBS, 100 μg/mL streptomycin, 100 units/mL penicillin, 12.5 units/mL nystatin and 2 mM L-glutamine. Cells were grown at 37°C; 5% CO_2.

Cell viability experiment

The assay was performed as previously reported in Gnaim, S. et al, Angew. Chem. Int. Ed. 2018, 57, 9033-9037. Briefly, HEK-293-WT and HEK-293-LacZ cells (15,000 cells/250 μL/well) were seeded in 250 μL of DMEM in 24 well corning clear bottom plates. Following 24 hours' incubation, 250 μL of serial dilutions (200 uM -0.2 nM) of compounds were added to each well (final concentration 100 μM - 0.1 nM). Number of viable cells was counted by a Z1 Coulter Counter® (Beckman Coulter®) following 48 h of incubation at 37°C, 5% CO_2.The viability of the cells was unharmed by all compounds at highest concentrations.

Protocol of chemiluminescence microscopy imaging of β-galactosidase activity

Chemiluminescence images were acquired using Olympus LV200 inverted microscope fitted with an EMCCD camera (Hamamatsu C9100-13). HEK-293-LacZ stable cells (amsbio SC003) and HEK-293-WT cells (control) were grown on 35 mm glass bottom petri dishes at 37°C for 24 h. Cell culture medium was changed to Molecular Probes® Live

Cell Imaging Solution containing 50 μM of probe **2** and 50 μM of **TMCD-FITC**. Cells were incubated for another 20 minutes at 37 °C. Thereafter, images were recorded with 5 minutes exposure time.

Protocol of BioSpace chemiluminescence imaging in living cells

HEK-293-WT and HEK-293-LacZ cells (25,000 cells/well) were seeded in 96 well corning clear bottom plates one day before treatment. Medium was removed and cells were washed with culture medium for 3 times and were treated 10 μM of probe **2** and 50 μM of **TMCD-FITC** and imaged by BioSpace Lab PhotonIMAGER™. Experiment was performed in triplicates.

Ethics Statement

All animal procedures were performed in compliance with Tel Aviv University, Sackler School of Medicine guidelines and protocols approved by the Institutional Animal Care and Use Committee.

Intravital non-invasive chemiluminescence imaging of probe 5 activation in mice

To induce acute inflammation, 1 mL (0.1 mg/mL) of LPS (Lipopolysaccharides from Escherichia coli 055:B5, Sigma) was injected into the peritoneal cavity (i.p.) of Balb/c mice. A second control group of mice was injected i.p. with 1 mL of PBS. Four hours later, both mice groups were additionally injected i.p. with 100 μL of 100 μM of probe **5** or100 μL of 100 μM of probe **5** and 300 μM of **TMCD-Cy5**. Twenty minutes later, mice were anesthetized using ketamine (100 mg/kg) and xylazine (12 mg/kg), and imaged by

BioSpace Lab PhotonIMAGER™. Activated probe **5** chemiluminescence signal was quantified as total signal of photons/exposure time (sec). Data is expressed as mean ± S.D.

^{31}P-NMR titration study

To determine the complexation constant of **TMCD** with probe **1**, titration experiment was carried out directly in an NMR tube. The kinetic of the complexation reaction was studied by ^{31}P-NMR spectroscopy. Typical procedure: 5 mg of the appropriate probe **1** was dissolved in 1000 µL D_2O. The latter solution was divided to two 500 µL solutions, A and B. Solution A was transferred to an NMR tube, and to solution B was added 50 mg of **TMCD**. The spectra were measured after the addition of various amounts of solution B (17, 33, 65, 129, 257, 500 µL) to solution A.

^{1}H-NMR crystallization experiment

The crystallization experiment was carried out by typical crystallization protocol. 960 mg of **TMCD** was dissolved in 950 µL of water and 50 µL of DMF, then 80 mg probe **2** was added to the solution. The solution was kept at dark environment at room temperature. After 7 days a white crystals was formed.

MS spectra of TMCD, TMCD-FITC and TMCD-Cy5:

MS analysis of **TMCD**:

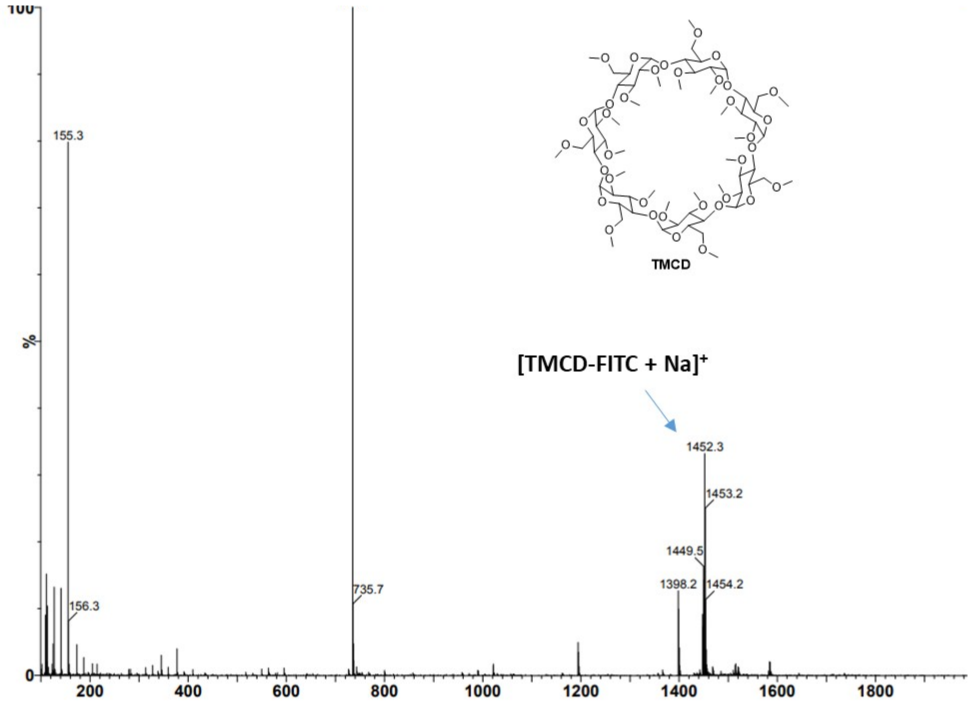

MS analysis of **TMCD-FITC**:

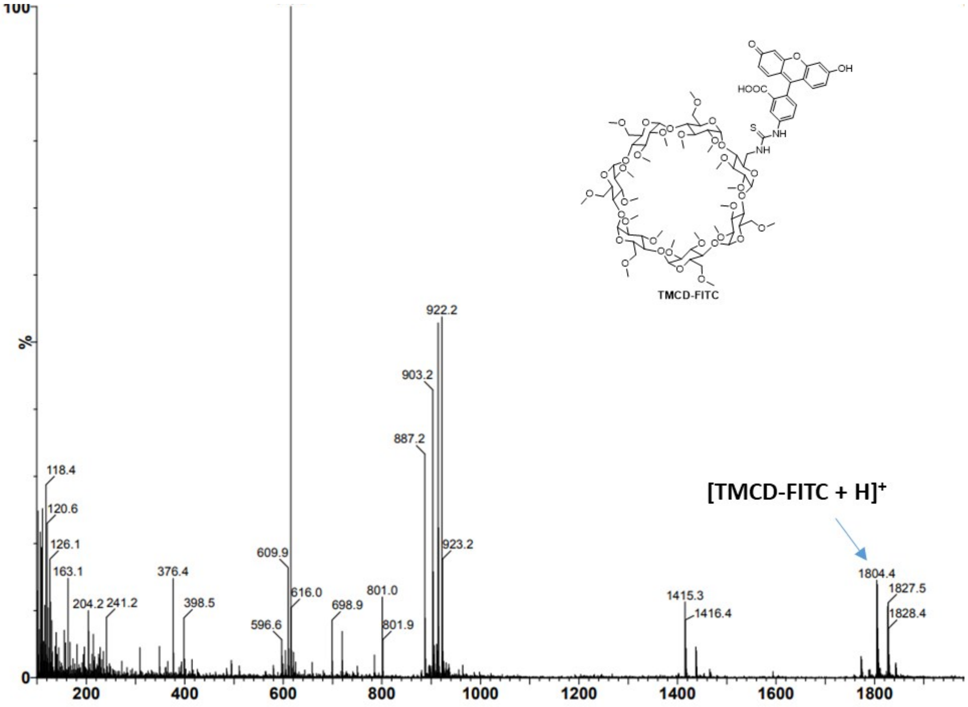

MS analysis of **TMCD-Cy5**:

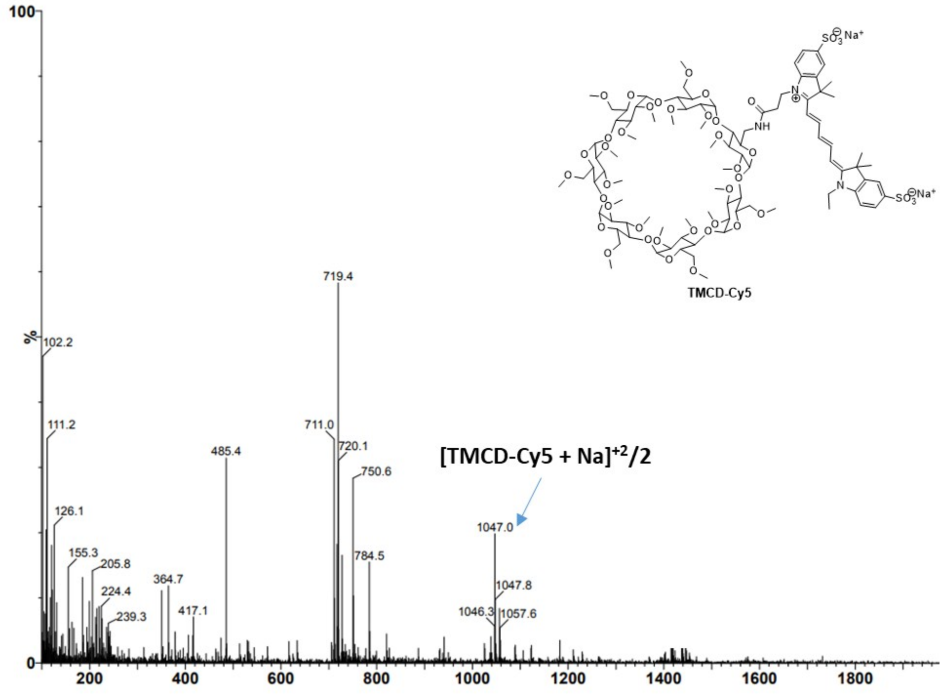

^{1}H-NMR spectra of probes 1-5, TMCD-FITC and TMCD-Cy5:

Compound 1:

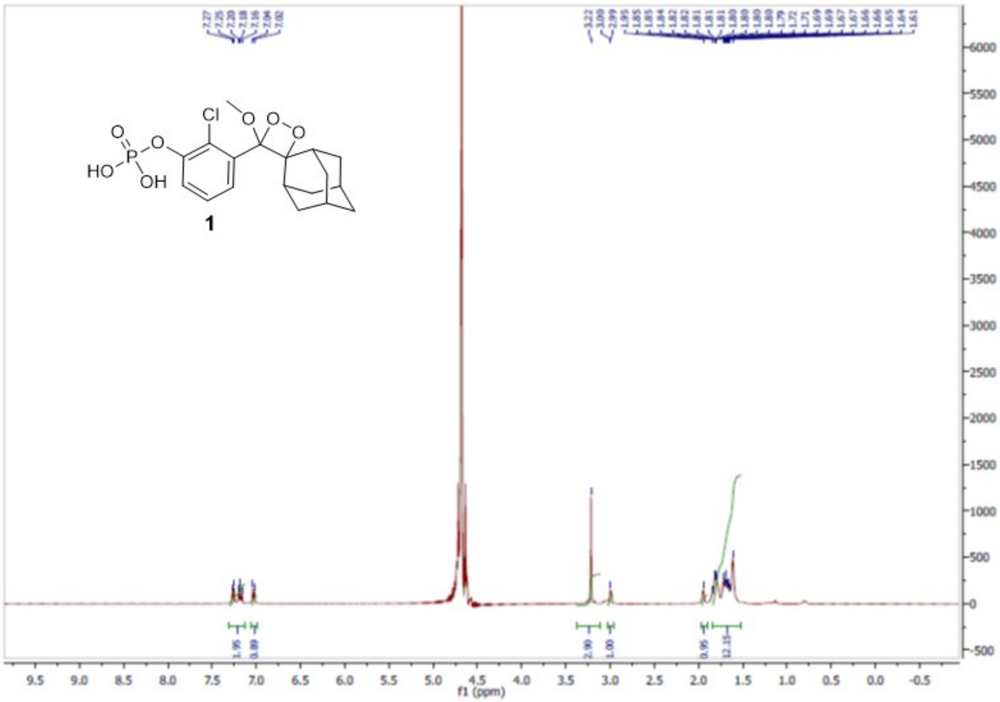

Compound 2:

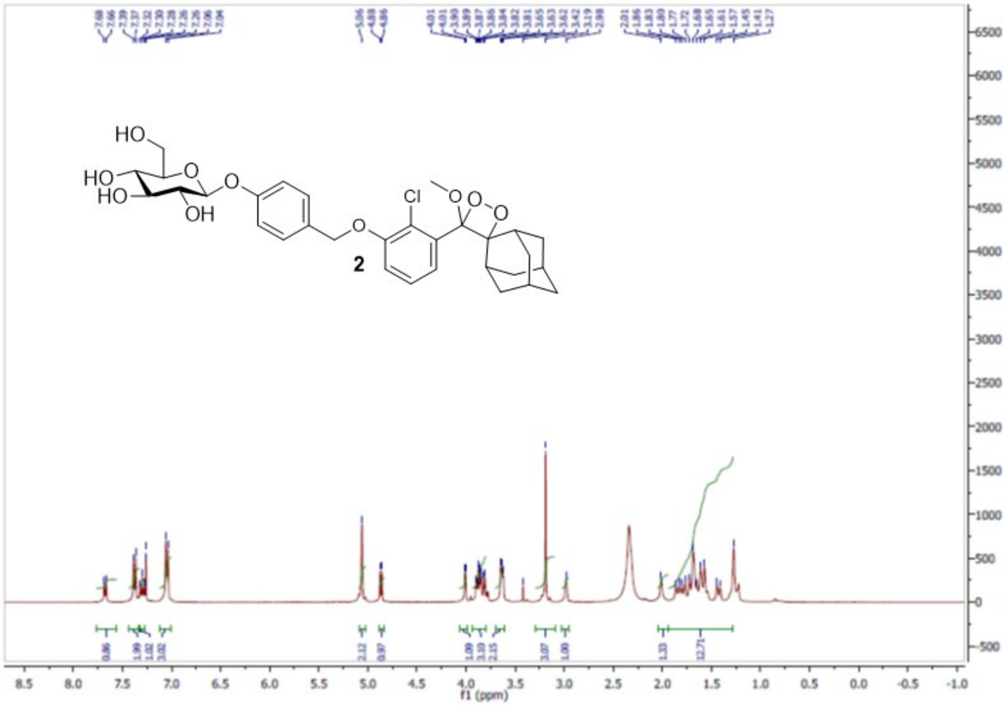

Compound 2c:

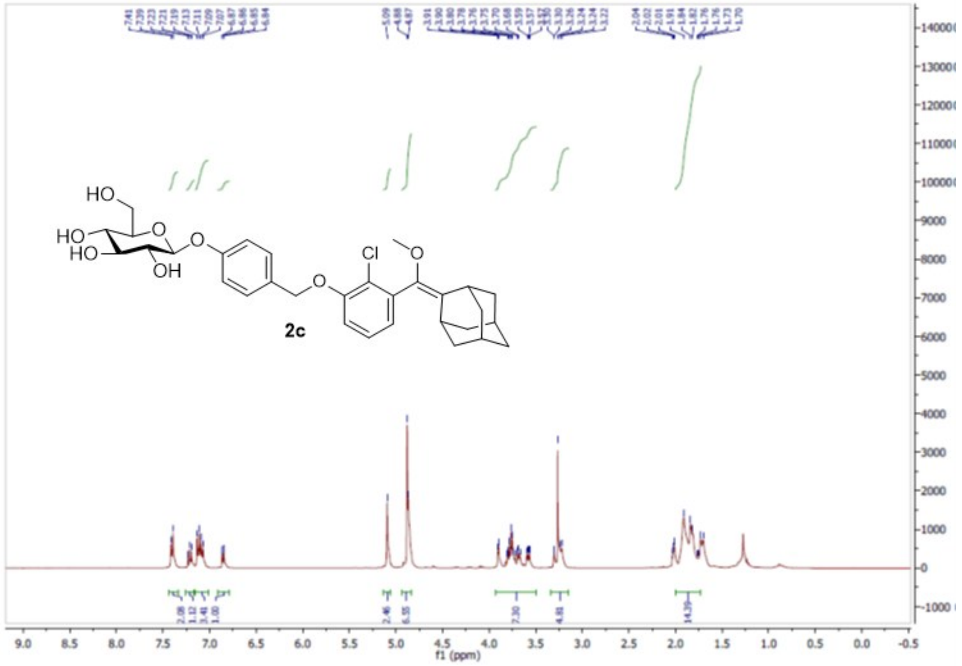

Compound 3:

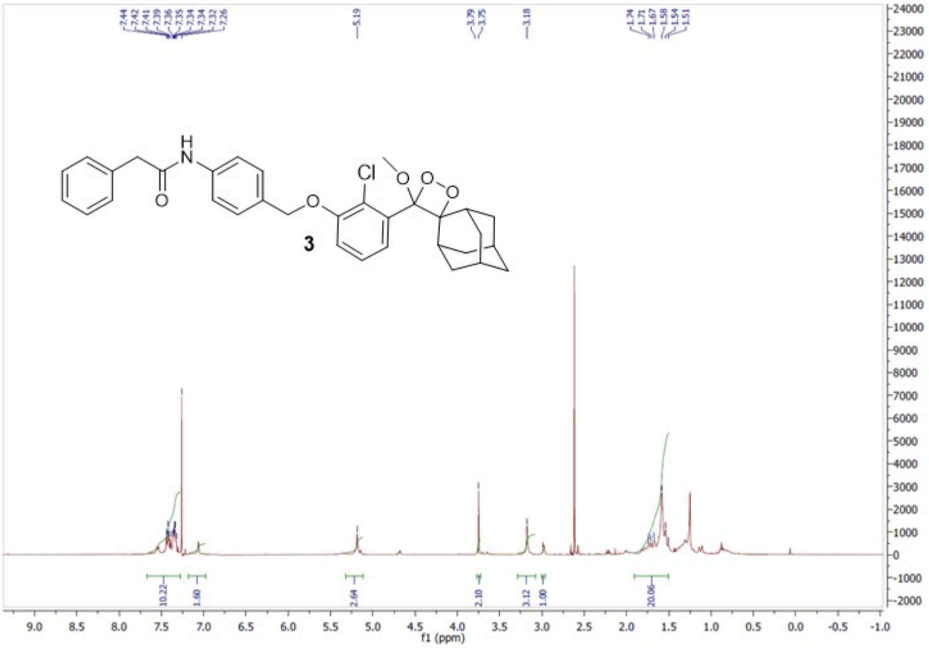

Compound 4:

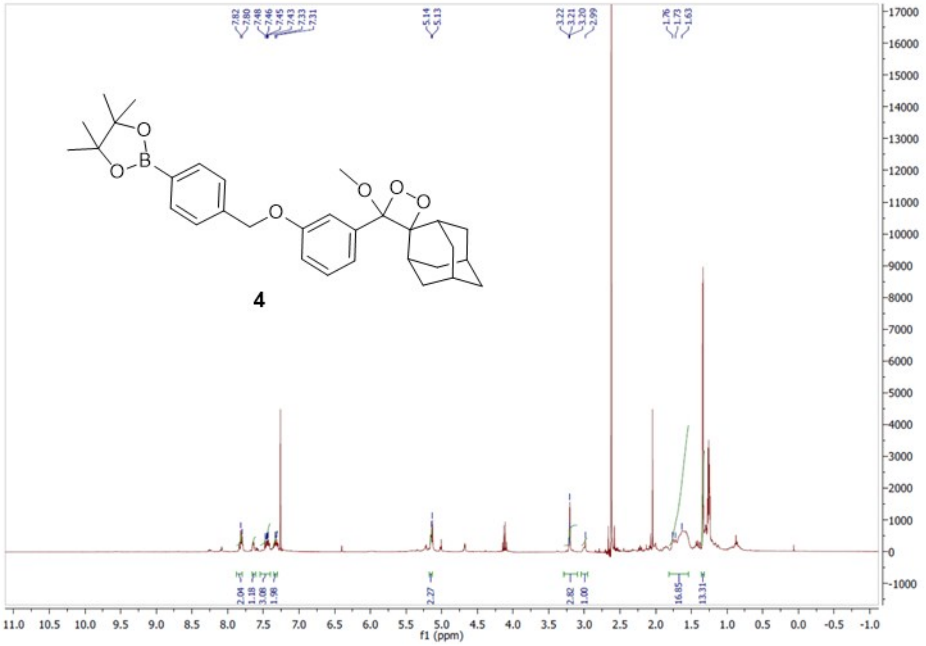

Probe 5:

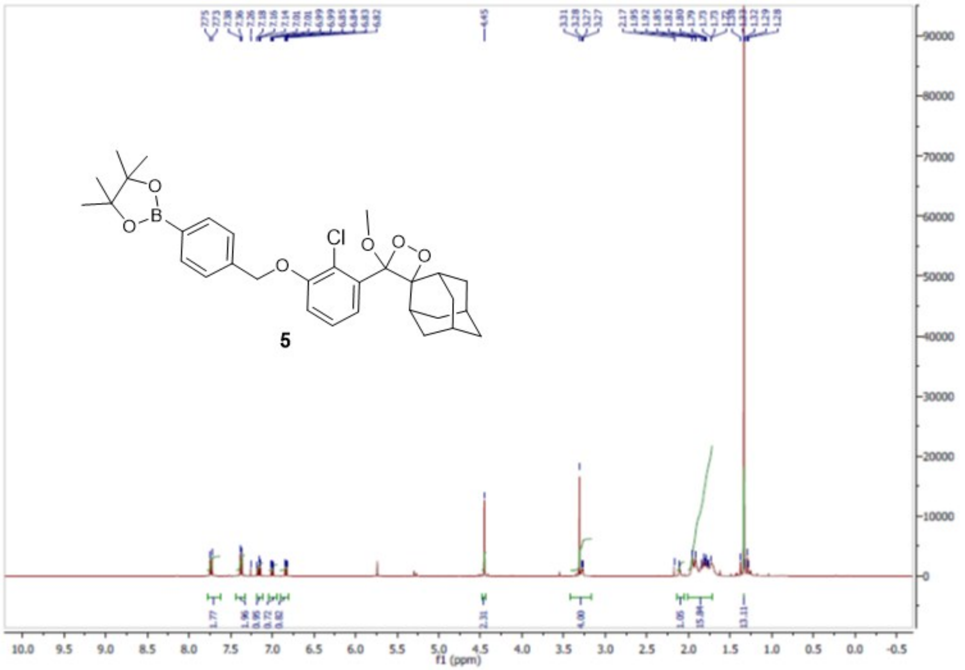

TMCD-FITC:

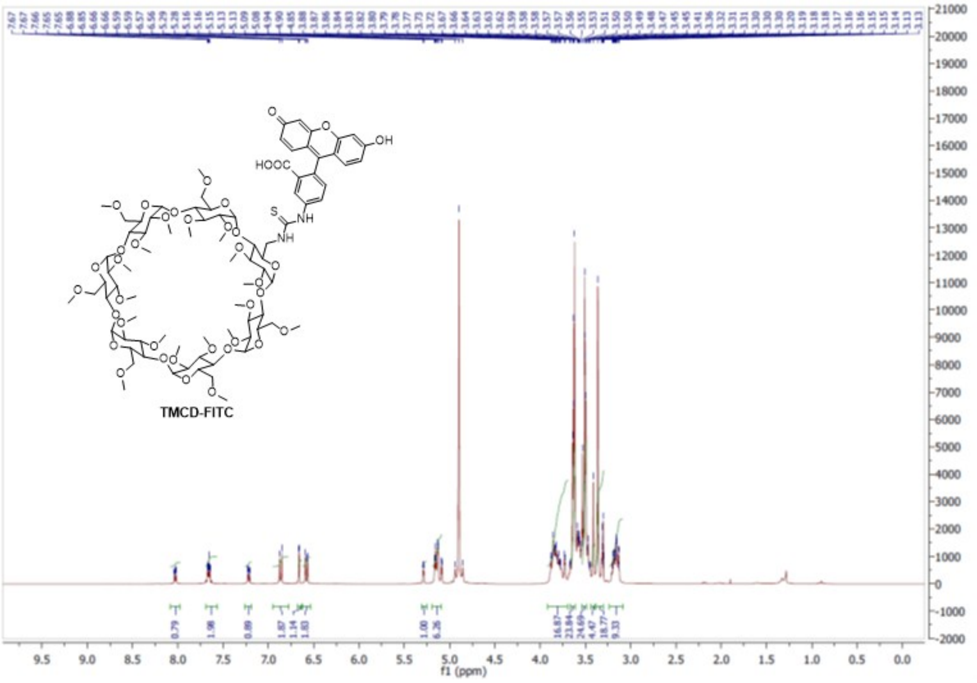

TMCD-Cy5:

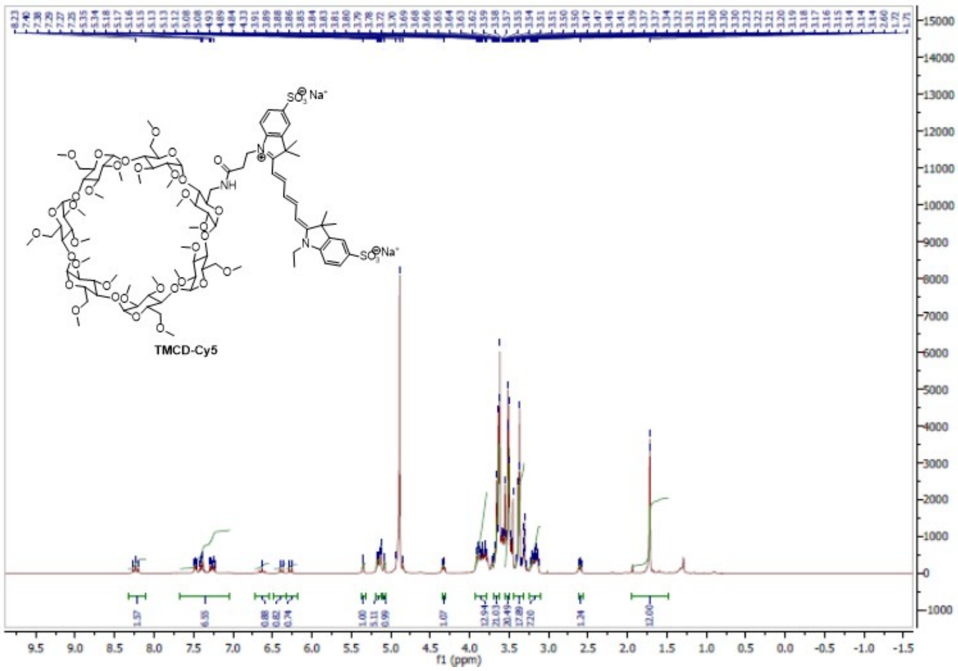

References:

1. Green, O.; Eilon, T.; Hananya, N.; Gutkin, S.; Bauer, C. R.; Shabat, D. *ACS Cent. Sci*. **2017**, 3, 349-358.
